# The E3 ubiquitin ligase TRIP12 participates in cell cycle progression and chromosome stability

**DOI:** 10.1038/s41598-020-57762-9

**Published:** 2020-01-21

**Authors:** D. Larrieu, M. Brunet, C. Vargas, N. Hanoun, L. Ligat, L. Dagnon, H. Lulka, R. M. Pommier, J. Selves, B. E. Jády, L. Bartholin, P. Cordelier, M. Dufresne, J. Torrisani

**Affiliations:** 1 Université de Toulouse, INSERM, Université Toulouse III-Paul Sabatier, Centre de Recherches en Cancérologie de Toulouse, Toulouse, France; 2 Université de Lyon, Université Claude Bernard Lyon 1, INSERM 1052, CNRS 5286, Centre Léon Bérard, Centre de recherche en cancérologie de Lyon, Lyon, 69008 France; 3 Laboratoire de Biologie Moléculaire Eucaryote du CNRS, UMR5099, Centre de Biologie Intégrative, Université Toulouse III-Paul Sabatier, Toulouse, Cedex 9, France

**Keywords:** Cell growth, Cancer genomics

## Abstract

Several studies have linked the E3 ubiquitin ligase TRIP12 (Thyroid hormone Receptor Interacting Protein 12) to the cell cycle. However, the regulation and the implication of this protein during the cell cycle are largely unknown. In this study, we show that TRIP12 expression is regulated during the cell cycle, which correlates with its nuclear localization. We identify an euchromatin-binding function of TRIP12 mediated by a N-terminal intrinsically disordered region. We demonstrate the functional implication of TRIP12 in the mitotic entry by controlling the duration of DNA replication that is independent from its catalytic activity. We also show the requirement of TRIP12 in the mitotic progression and chromosome stability. Altogether, our findings show that TRIP12 is as a new chromatin-associated protein with several implications in the cell cycle progression and in the maintenance of genome integrity.

## Introduction

TRIP12 (Thyroid Hormone Receptor Interacting Protein 12) is a 225 kDa HECT (Homologous to the E6-AP Carboxyl Terminus) domain-containing E3 ubiquitin ligase. It is also known as ULF (Ubiquitin Ligase for ARF)^1^ and UFD4 (Ubiquitin Fusion Degradation) in S. cerevisae^2^. TRIP12 contains protein interacting WWE (tryptophan and glutamate conserved residues) and β-ARM (β-Armadillo) domains^3^. Several functions are attributed to TRIP12. For instance, it ubiquitinates APP-BP1 (Amyloid Precursor Protein-Binding Protein 1) and BAF57 (Brg1-Associated Factor 57) turn over and is therefore described as a sensor of SWI/SNF (SWItch/Sucrose Non-Fermentable) complex integrity^4,5^. TRIP12 is also known to control the histone ubiquitination after DNA breakage^6^ and to contribute to p14/ARF (Alternate Reading Frame) degradation in response to DNA damage^7^. TRIP12 ensures the proteolysis of ASLX1 (Additional Sex Combs Like 1, Transcriptional Regulator), a regulatory component of the ubiquitin hydrolase BAP1 (BRCA1 Associated Protein 1)^8^. We showed that TRIP12 is implicated in the proteasome-mediated degradation of the transcription factor PTF1a (Pancreas specific Transcription Factor 1a) that plays an important role in the maintenance of the acinar phenotype in human pancreas^9^. TRIP12 is essential for cell viability as a homozygous mutation that disrupts the ubiquitin ligase activity leads to murine embryonic lethality^10^. Importantly, TRIP12 plays an important role in the cell cycle. For instance, ^Trip12 mt/mt^ ES cells display reduced growth with increased expression of the p16/CDKN2A gene and an accumulation in G_2_/M phase^10^. Moreover, by controlling the tumor suppressor protein p14/ARF, TRIP12 impacts TP53 protein level, a major regulator of the cell cycle^11^. TRIP12 expression is altered in several types of cancer, such as breast and pancreatic cancer^11^. High expression of TRIP12 is associated with poor prognosis in hepatocellular carcinoma after surgical resection^12^. TRIP12 is involved in radio-sensitization of head and neck squamous carcinoma^13^. TRIP12 mutations are also related to autism disorders^14^ and intellectual disability^15^.

Cell cycle progression is governed by sequentially organized events. Mitogen signaling pathways impose to the cell the exit of G_1_ phase to duplicate its genome. The licensing of replication (Minute Chromosome Maintenance) to form the pre-replication complex (pre-RC). In S phase, the origins firing is dependent of the assembly of other proteins and the activity of CDKs (Cyclin Dependent Kinase). A multitude of additional factors are then recruited such as RPA (Replication Protein A), PCNA (Proliferating Cell Nuclear Antigen) and polymerases to constitute the fully functional replisome^16^. DNA replication is spatio-temporally ordered. Replication of euchromatin regions occurs in early S phase whereas heterochromatin regions in late S phase^17^. Subsequently, cells enter in a transition phase G_2_ that ensures the complete DNA replication. In late G_2_ phase, the initiation of mitosis is promoted by a complex comprising of the CYCLIN B1 and the Cyclin-Dependent Kinase 1 (CDK1)^18^. This complex is inhibited by the phosphorylation of Tyrosine 15 residue on CDK1 by the WEE1 kinase^19^. Chromatin condensation, separation of duplicated centrosomes and the recruitment of proteins to the kinetochores, all mark the prophase. The resulting release of chromosomes activates the Spindle Assembly Checkpoint (SAC) that controls the proper segregation of chromosomes and inhibits the Anaphase Promoting Complex/Cyclosome (APC/C) complex. In metaphase, the correct attachment of chromosomes to the kinetochores inhibits the SAC allowing for progression and ending of mitosis^20–22^. Mitotic dysfunction leads to chromosomal instability and aneuploidy favoring the initiation of cancer^23^. A prolonged activation of the SAC can provoke cell death or mitotic exit without separation of sister chromatids; named mitotic slippage. Improper separation of sister chromatids (anaphase bridges) induces the formation of micronuclei, which can be at the origin of DNA damages^24^. The succession of the different cell cycle phases requires a perfectly coordinated expression and activation of multiple proteins. This coordination is ensured by a multi-layer regulation including transcription, mRNA degradation, translation, post-translation modification and protein degradation^25^. For instance, protein expression of CYCLIN A begins in S phase, peaks during G_2_ phase and dramatically declines in mitosis. It combines a tight coordination of transcription, mRNA translation and protein degradation.

Among the proteins that participate in cell cycle regulation, a significant proportion interacts with chromatin to modulate, for instance, the transcription (*i.e*.: transcription factors) and the chromatin structure (i.e.: histones, chromatin remodeling complexes, condensin). These proteins can bind to chromatin by recognition to a specific DNA consensus sequence *via* different DNA-binding domains. (*i.e*.: β-Helix-Loop-Helix, zinc fingers) but also *via* intrinsically disordered regions (IDR). IDRs are protein domains that lack a stable 3D structure under physiological conditions. IDRs can be predicted from the amino acid sequence according to their physicochemical properties^26^. They interact with DNA in a nucleotide sequence-independent manner, thereby modifying chromatin structure and regulating gene expression^27^.

Our findings expose for the first time that TRIP12 protein expression is tightly regulated during cell cycle, and, that TRIP12 interacts with euchromatin through a new functional N-terminal domain. By means of this chromatin interaction, we further propose that TRIP12 participates in mitotic entry by controlling duration of DNA replication. We further demonstrate that TRIP12 is implicated in mitotic progression and in chromosome stability.

## Results

### TRIP12 expression is regulated during the cell cycle

**Figure 1:**
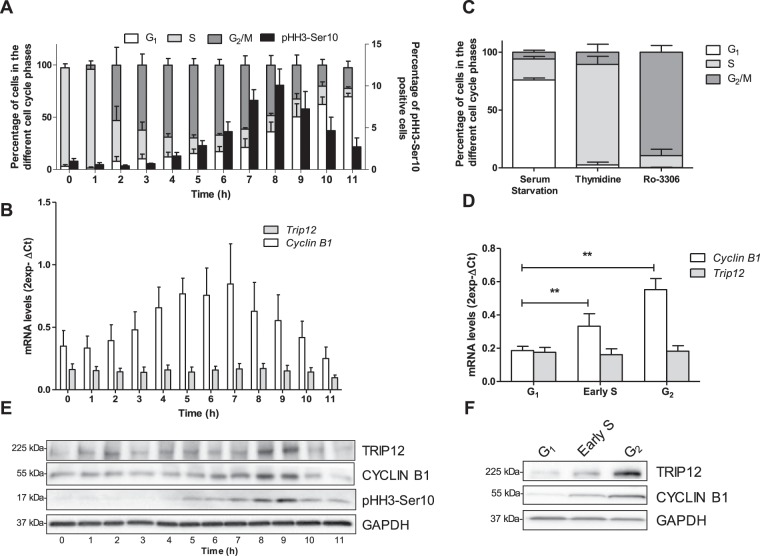
TRIP12 expression is regulated during the cell cycle. (**A**) Distribution of cells in the different phases of the cell cycle and the percentage of pHH3-Ser10 positive cells (black bars) were assessed by flow cytometry in HelaS3 cells arrested in early S phase using a double thymidine block and released in fresh medium for the indicated times. The bars represent the mean ± SEM obtained from three different experiments. (**B**) Expression level of *Trip12* and *Cyclin B1* mRNA was measured by RT-qPCR in HelaS3 cells arrested in early S phase using a double thymidine block and released in fresh medium for the indicated times. The bars represent the mean ± SEM of mRNA levels (expressed as 2^exp-ΔCt^) obtained from three different experiments. (**C**) Distribution of HelaS3 cells in the different phases of the cell cycle after serum starvation, double thymidine block and RO-3306 treatments was determined by flow cytometry. The bars represent the percentage expressed as a mean ± SEM obtained from three different experiments. (**D**) Expression level of *Trip12* and *Cyclin B1* mRNA was measured by RT-qPCR in HelaS3 cells were arrested in G_1_, early S, and G_2_ phase using serum starvation, double thymidine block and Ro-3306, respectively. The bars represent the mean ± SEM of mRNA levels (expressed as 2^exp-ΔCt^) obtained from three different experiments. ** indicates a p value < 0.01. (**E**) TRIP12, CYCLIN B1 and pHH3-Ser10 level was measured by Western blot analysis in HelaS3 cells arrested in early S phase using a double thymidine block and released in fresh medium for the indicated times. GADPH protein level was used as loading control. Images were obtained from the same experiment and representative of three different experiments. (**F**) TRIP12 and CYCLIN B1 levels were measured by Western blot analysis in HelaS3 cells arrested in G_1_, early S and G_2_ phase using serum starvation, double thymidine block and Ro-3306 treatments, respectively. GADPH protein level was used as loading control. Images were obtained from the same experiment and representative of three different experiments.

The E3 ubiquitin ligase TRIP12 was shown to control the expression of important regulators of the cell cycle progression. However, the regulation of TRIP12 during the cell cycle is still unknown. To address this issue, HelaS3 cells were arrested at the G_1_/S boundary and released in the cell cycle **(**Fig. 1A**)**. The experiment showed a maximal percentage (10.1%) of cells in early mitosis 8 h after release and a maximal percentage of cells in G_1_ phase (69.6%) 11 h after release. The level of *Trip12* mRNA was measured and did not fluctuate during the cell cycle kinetics **(**Fig. 1B**)**. As a control, we measured the expression of *Cyclin B1* mRNA level that is known to be up-regulated in early S phase until G_2_ phase^28^. Similarly, *Trip12* mRNA level did not vary in G_1_-, early S- and G_2_-phase-enriched cell populations unlike *Cyclin B1* mRNA **(**Fig. 1C,D**)**, that confirms our results **(**Fig. 1B**)**.

Next, TRIP12 protein level was measured following the same kinetics **(**Fig. 1E**)**. Present in S phase, TRIP12 expression gradually increases to reach a maximal expression in G_2_ phase and mitosis. Interestingly, TRIP12 protein level decreased to reach a minimal expression when cells enter in G_1_ phase. TRIP12 protein level was also measured in enriched cell populations in G_1_, early S, and G_2_ phase **(**Fig. 1F**)**. As expected, TRIP12 protein was barely detected in G_1_ phase, appears in early S phase to reach a maximal expression in G_2_ phase. These results clearly demonstrate that TRIP12 protein expression varies throughout the cell cycle while its mRNA remains at a constant level and, they suggest that TRIP12 protein is degraded during G_1_ phase, or *Trip12* mRNA translation is tightly regulated, or a combination of both.

### TRIP12 is a cell cycle-regulated nuclear protein associated with chromatin

**Figure 2:**
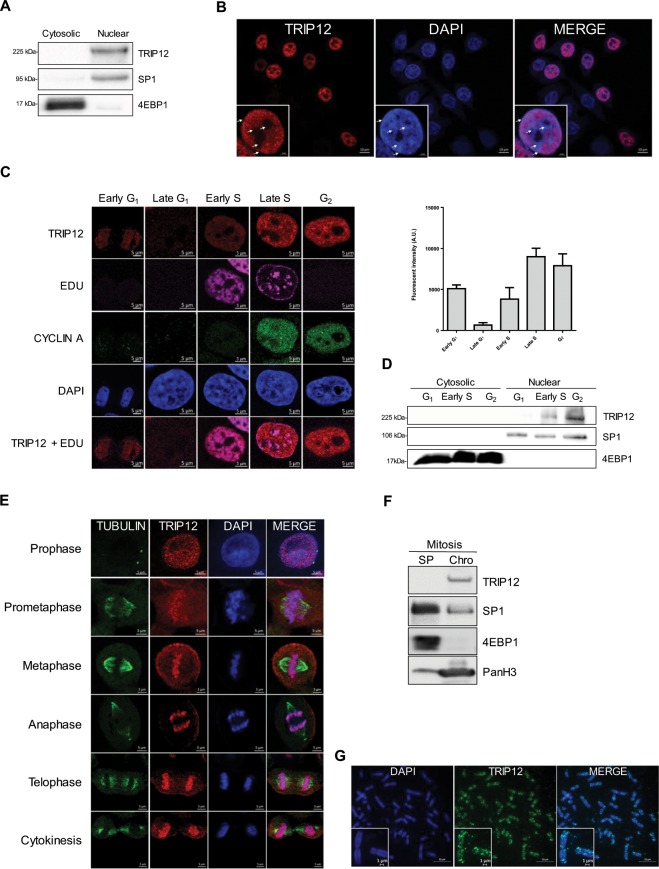
TRIP12 is a cell cycle-regulated nuclear protein associated with chromatin. (**A**) TRIP12 protein level in cytosolic and nuclear fractions of HelaS3 cells was measured by Western blot analysis. SP1 and 4EBP1 protein levels were used as purity controls of subcellular fractions. Images were obtained from the same experiment and representative of three different experiments. (**B**) TRIP12 subcellular localization in HelaS3 cells was determined by immunofluorescence using TRIP12 antibody (Sigma). The inset represents TRIP12 nuclear localization at a higher magnification. Nuclei were counterstained with DAPI. The white arrows indicate the peri-nucleolar and peri-nuclear heterochromatin regions. (**C**) TRIP12 nuclear expression in HelaS3 cells in the different phases of the cell cycle was determined by immunofluorescence using TRIP12 antibody (Sigma). Nuclei were counterstained with DAPI. Cells in G_1_ phase and G_2_ phase correspond to CYCLIN A/EDU nuclear negative cells and CYCLIN A nuclear positive/EDU negative cells, respectively. Cells in early G_1_ correspond to G_1_ cells with small oblong-shaped nucleus. Cells in early S and late S phase correspond to EDU positive cells/CYCLIN A negative and positive nuclear cells, respectively. The graph represents the mean TRIP12 expression (integrated density-background) ± SEM determined at least 200 cells using FIJI software. (**D**) Level of TRIP12 in cytosolic and nuclear protein fraction of G_1_-, early S- and G_2_-phase enriched HelaS3 cell populations was measured by Western blot analysis. SP1 and 4EBP1 protein levels were used as purity control of subcellular fractions. Images were obtained from the same experiment and representative of three different experiments. (**E**) TRIP12 and γ-TUBULIN localization in HelaS3 cells in prophase, prometaphase, metaphase, anaphase, telophase and cytokinesis were visualized by immunofluorescence. Nuclei were counterstained with DAPI. (**F**) TRIP12 expression in soluble protein (SP) and chromatin-bound (Chro) protein fractions (40 µg for each fraction) obtained from nocodazole-treated mitotic HelaS3 cells was measured by Western blot analysis. As mitotic cells do not have nuclear membrane, SP fraction contains cytoplasmic and nuclear soluble proteins. SP1, 4EBP1 and panH3 protein levels were used as purity control of the different fractions. Images were obtained from the same experiment. (**G**) TRIP12 localization on metaphasic chromosomes was visualized by immunofluorescence using anti-TRIP12 antibody (Sigma) after chromosome spreading of HelaS3 cells treated with Ro-3306 and released in the cell cycle for 45 min. DNA was counterstained with DAPI. The inset represents a magnification of TRIP12 localization on two individual chromosomes.

We tested whether TRIP12 cell-cycle regulation correlated with its sub-cellular localization. First, we first showed that TRIP12 is a nuclear protein but it is not present in all cells **(**Figs. 2A,B and S1A). The specificity of TRIP12 nuclear staining was verified (Fig. S1B). Interestingly, TRIP12 nuclear staining did not colocalize with dense DAPI regions that correspond to peri-nuclear and peri-nucleolar heterochromatin regions (Fig. 2B), suggesting that TRIP12 preferentially colocalizes with euchromatin regions. TRIP12 nuclear localization was assessed during the cycle of asynchronous HelaS3 cells. We found that TRIP12 is barely detectable in late G_1_ cells and appears in early S phase cells reaching a maximal detection in late S and G_2_ cells (Fig. 2C). TRIP12 absent-cells observed in Fig. 2B correspond to cells in G_1_ phase. It is known that euchromatin regions are replicated early during S phase and are stained homogenously after EDU incorporation. On the contrary, heterochromatin regions are replicated late in S phase and form punctiform staining with EDU. Interestingly, TRIP12 staining corresponds to DNA regions with homogenous and not punctiform EDU staining **(**Fig. 2C**)**, corroborating the localization of TRIP12 on euchromatin regions **(**Fig. 2B**)**. Cell cycle-dependent presence of TRIP12 in the nucleus was confirmed in G_1_-, early S- and G_2_-phase enriched cell populations (Figs. 2D and S1C). We clearly visualized TRIP12 association with chromatin in mitotic cells in which chromatin is subject to condensation and decondensation **(**Fig. 2E**)**. TRIP12-chromatin association was also confirmed by chromatin-bound protein fractionation **(**Fig. 2F**)**. The colocalization with chromatin during metaphase was also observed using a HA-tagged TRIP12 construct (Fig. S2A). Chromosome spreading experiments revealed that TRIP12 colocalizes with chromatin on full-length chromosomes **(**Figs. 2G and S2B).

### TRIP12 is associated with chromatin through a N-terminal intrinsically disordered region

**Figure 3:**
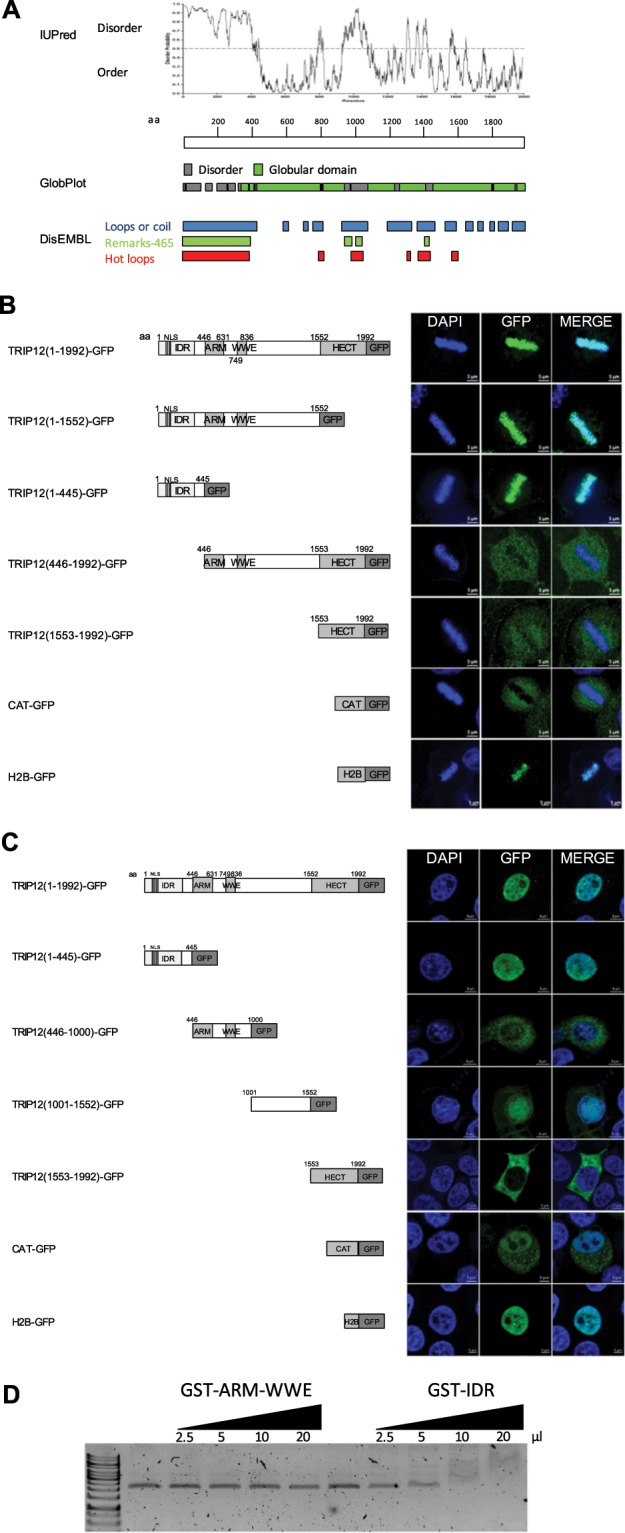
TRIP12 is associated with chromatin through a N-terminal intrinsically disordered region. (**A**) Prediction of intrinsically disordered and ordered regions in TRIP12 protein sequence (Protein accession number NP_004229.1) was determined using IUPred, GlobPlot and DisEMBL software. aa stands for amino acids. (**B**) GFP-fusion protein localization in metaphasic HelaS3 cells transfected with TRIP12 (1-1992), (1-1552), (1-445), (446-1992) and (1553-1992)-GFP constructs was visualized by immunofluorescence using an anti-GFP antibody. H2B-GFP and CAT-GFP constructs were used as controls. Nuclei were counterstained with DAPI. IDR stands for Intrinsically Disordered Region, NLS for Nuclear Localization Signal, ARM for β-Armadillo domain, WWE for tryptophan and glutamate enriched domain and HECT for Homologous to the E6-AP Carboxyl Terminus. Numbers indicate amino acids (aa) position. (**C**) GFP-fusion protein localization in interphasic HelaS3 cells transfected with TRIP12 (1-1992), (1-445), (446-1000), (1001-1552) and (1553-1992)-GFP constructs was analyzed by immunofluorescence using an anti-GFP antibody. H2B-GFP and CAT-GFP constructs were used as controls. Nuclei were counterstained with DAPI. IDR stands for Intrinsically Disordered Region, ARM for β-Armadillo domain, WWE for tryptophan and glutamate enriched domain and HECT for Homologous to the E6-AP Carboxyl Terminus. Numbers indicate amino acids (aa) position. (**D**) Interaction of increasing amounts of GST-TRIP12-ARM-WWE(446-1000) and GST-IDR(1-325) with naked plasmid DNA visualized after migration on agarose gel.

We identified several IDRs in the TRIP12 sequence that could potentially act as chromatin interacting-domains, notably one in the N-terminus located between the amino-acids 1 and 440 **(**Fig. 3A**)**. An analysis in metaphasic cells showed that TRIP12 colocalization with chromatin is mediated by its N-terminal IDR. Whereas the deletion of the C-terminal HECT domain (TRIP12(1-1552)-GFP) does not affect the capacity of the fusion protein to colocalize with chromatin, the deletion of the IDR (TRIP12(446-1992)-GFP) abolishes it **(**Fig. 3B**)**. In parallel, the subcellular localization of full-length TRIP12-GFP and four TRIP12-GFP deletion constructs in cells in interphase led us to conclude that, similar to endogenous TRIP12, TRIP12(1-1992)-GFP localizes exclusively in the nuclear compartment. More importantly, the TRIP12(1-445)-GFP localizes in the nucleus demonstrating that the N-terminal IDR is sufficient for the nuclear localization of TRIP12; suggesting that this region contains a nuclear localization signal **(**Fig. 3C**)**. Moreover, IDR alone can ensure a physical direct interaction with naked DNA **(**Fig. 3D**)** and the localization of GFP on full-length metaphasic chromosomes (Figs. S2A and S3B). The colocalization of TRIP12 and its IDR with chromatin was confirmed in living cells (Figs. S3C). A more precise analysis of the IDR reveals that the region between the amino acids 107 and 325 ensures the interaction of TRIP12 with chromatin (Fig. S3D,E). Interestingly, cells that express a high level of the IDR construct display characteristic features of cells in prophase with condensed chromosomes covering the inner face of the nuclear membrane (Fig. S3F) even if they are not pHH3-Ser10 positive (Fig. S3G, bottom panel) as they should be a normal cell (Top panel). This suggests that TRIP12 *via* its IDR could participate in chromatin compaction.

Altogether, our experiments identify a new functional domain of TRIP12 that permits the association of the protein with chromatin.

### A deregulated expression of TRIP12 affects cell division and mitotic entry

**Figure 4:**
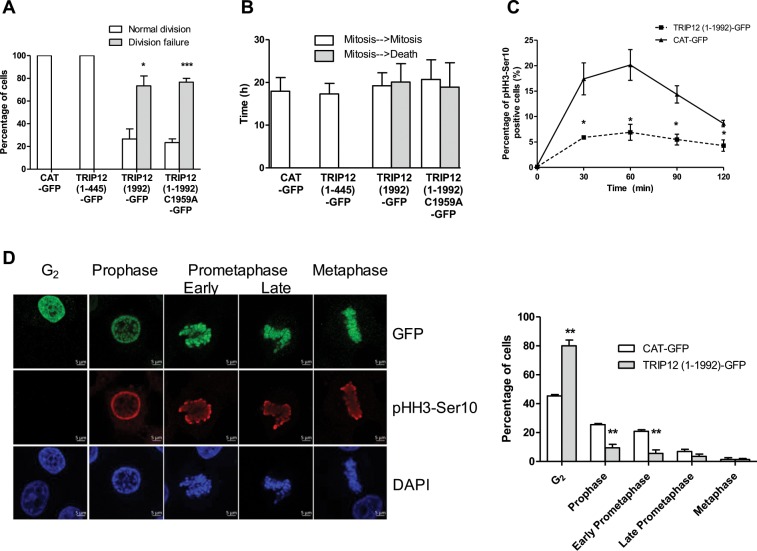
An over-expression of TRIP12 affects cell division by delaying mitotic entry. (**A**) The graph represents the percentage ± SEM of GFP-cells that overcome at least two cell divisions or die over a 48h-period. * and *** indicate a p value < 0.05 and < 0.001, respectively. (**B**) The graph represents the average duration ± SEM in interphase between two mitoses (white bars) and between mitosis and cell death (grey bars) in the different GFP positive cells. (**C**) Percentage of pHH3-Ser10 positive HelaS3 cells transfected with TRIP12(1-1992)-GFP or control CAT-GFP constructs and arrested by Ro-3306 treatment was assessed by flow cytometry after release in inhibitor free medium every 30 min for 2 h. The graph represents the mean ± SEM of three different experiments. * indicates a p value < 0.05. (**D**) Proportion of cells in G_2_, prophase, early prometaphase, late prometaphase and metaphase in HelaS3 cells previously transfected with TRIP12(1-1992)-GFP or control CAT-GFP constructs was determined by immunofluorescence using anti-GFP (top panel) and anti-pHH3-Ser10 (middle panel) antibodies. Forty-eight hours after transfection, cells were arrested in G_2_ phase by a Ro-3306 treatment and released in inhibitor free medium for 30 min. GFP-positive cells that were negative for pHH3-Ser10 staining were considered in G_2_ phase. DNA was counterstained with DAPI. The graph represents the percentage of cells in the different phases expressed as mean ± SEM obtained from a minimum of 300 cells of three different experiments. ** indicates a p value < 0.01.

Given that TRIP12 expression and chromatin association are cell cycle dependent, we tested the consequences of a TRIP12 ectopic over-expression on cell fate during 48 h. We found that only 26.7% (8 out of 30) of TRIP12(1-1992)-GFP cells undergo two cell divisions or more **(**Figs. 4A and S4A,B) with a duration in interphase of 19.2 h **(**Fig. 4B**)**. The remaining 73.7% (22 out of 30) fail to achieve a single division and die **(**Figs. 4A and S4A,B**)**. Remarkably, this death occurs at a time (20.1 h) that mitosis should occur **(**Fig. 4B**)** suggesting that TRIP12 overexpression is deleterious for the mitotic division. This inhibitory effect on cell division was also observed after expression of an inactive mutant as well as several TRIP12 protein domains with the exception of the IDR of TRIP12 **(**Figs. 4A,B and S4A–E) suggesting that the mitotic inhibitory effect of TRIP12 is independent of chromosome binding and catalytic activity. We further demonstrated that TRIP12 over-expression diminishes the percentage of cells that enter in mitosis **(**Fig. 4C**)**. When we focused on TRIP12(1-1992)-GFP expressing cells that undergo cell divisions (Fig. S4A), we noticed that TRIP12 over-expression significantly delays the entry into prophase 30 min after Ro-3306 release **(**Fig. 4D, Graph).

**Figure 5:**
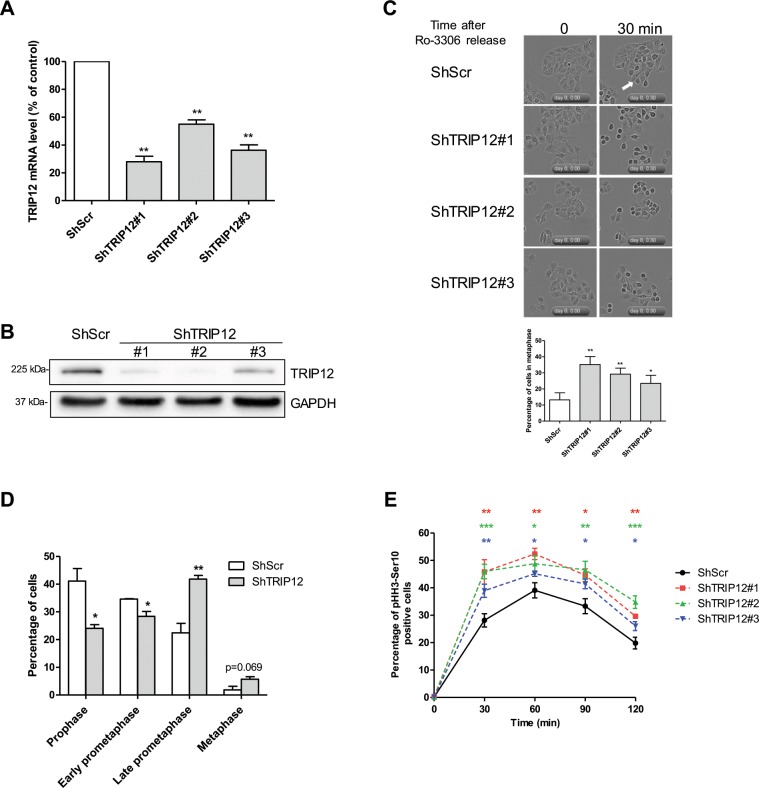
A TRIP12 depletion accelerates mitotic entry. (**A**) Expression of *Trip12* mRNA was determined by RT-qPCR in HelaS3 cells transduced with 3 shRNAs directed against *Trip12* mRNA (ShTRIP12) or scramble control shRNA (ShScr). *β-Actin*, *Gapdh* and *Cyclophilin* A mRNA level was used for normalization. Results are the mean ± SEM obtained from three different experiments and expressed as percentage of expression compared to ShScr control cells. ** indicates a p value < 0.01. (**B**) Expression of TRIP12 protein in HelaS3 ShTRIP12 and ShScr cells was determined by Western blot analysis. GAPDH protein level was used as loading control. Images were obtained from the same experiment and representative of three different experiments. (**C**) Percentage of cells in metaphase in TRIP12-depleted (ShTRIP12#1, #2 and #3) and control (ShScr) HelaS3 cells arrested by a Ro-3306 treatment and released in the cell cycle by medium replacement (t = 0) was determined by cell live microscopy. The graph represents the percentage of cells in metaphase phenotype expressed as mean ± SEM relatively to total number of cells. A cell was considered in metaphase phenotype when the equatorial plate was microscopically visualized. A representative cell in metaphase phenotype is indicated by the white arrow. The quantification was performed from 9 different fields from three different experiments representing an average of 650 analyzed cells. * and ** indicates a p value < 0.05 and 0.01, respectively. (**D**) Proportion of cells in prophase, early prometaphase, late prometaphase and metaphase in TRIP12-depleted (ShTRIP12#1) and control (ShScr) HelaS3 cells arrested by a Ro-3306 treatment and released in the cell cycle by medium replacement was determined by immunofluorescence using anti-pHH3-Ser10 antibody after 30 min. DNA was counterstained with DAPI. The graph represents the percentage of cells in the different steps of early mitosis expressed as mean ± SEM obtained from a minimum of 300 cells of three different experiments. * and ** indicate a p value < 0.05 and 0.01, respectively. (**E**) Percentage of pHH3-Ser10 positive cells in TRIP12-depleted (ShTRIP12) and control (ShScr) HelaS3 cells arrested in G_2_ phase by a Ro-3306 treatment and released in the cell cycle by medium replacement was determined by flow cytometry at the indicated time. Three different ShTRIP12 (#1, #2 and #3) were used for these experiments. The results represent the mean ± SEM obtained from four different experiments. *, ** and *** indicate a p value < 0.05, 0.01 and 0.001, respectively.

The inhibitory effect of TRIP12 over-expression on mitotic entry led us to investigate the consequences of a TRIP12 depletion on mitotic entry. Toward this end, TRIP12-depleted (ShTRIP12) and control (ShScr) cells were created using shRNAs **(**Fig. 5A,B**)**. After Ro-3306 release (30 min), we microscopically observed an accelerated mitotic entry in TRIP12-depleted cells **(**Fig. 5C**)** that was associated to a higher percentage of cells in late prometaphase and metaphase **(**Fig. 5D**)**. The accelerated mitotic entry was confirmed by flow cytometry-measurements of pHH3-Ser10 positive cells **(**Fig. 5E**)**. A normal mitotic entry of TRIP12-depleted cells was restored by a transient expression of TRIP12 or the catalytic mutant but not by an expression of the IDR **(**Fig. S5A**)**.

Altogether, these experiments reveal the important contribution of TRIP12 in mitotic entry as a deregulated expression of TRIP12 significantly impedes the proper setting of mitotic entry-regulatory mechanisms.

### A shortened duration of DNA replication explains the accelerated mitotic entry of TRIP12-depleted cells

**Figure 6:**
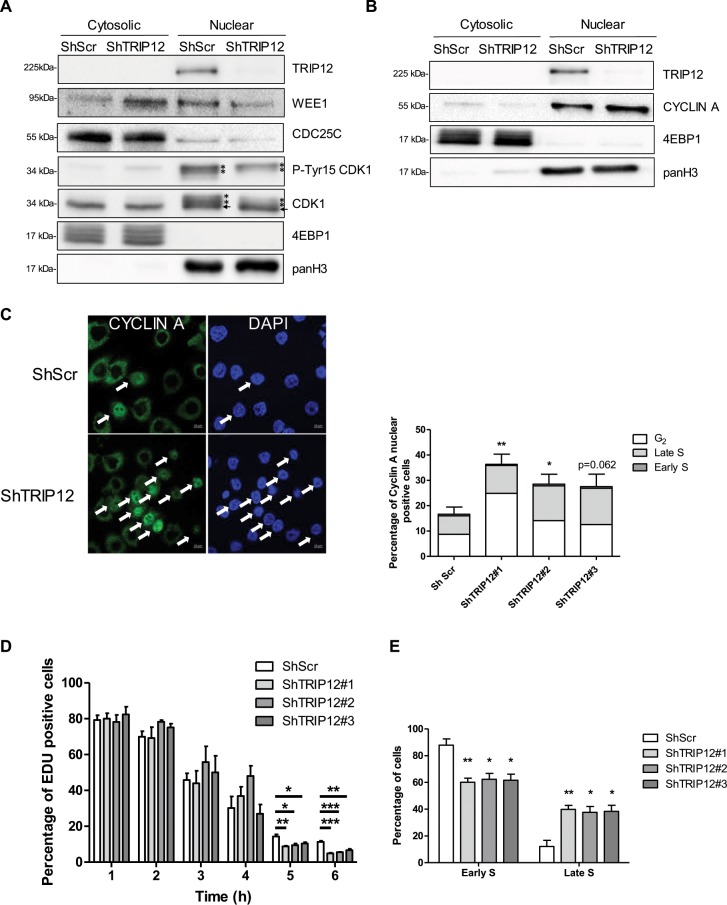
A shortened duration of DNA replication explains the accelerated mitotic entry of TRIP12-depleted cells. (**A**) TRIP12, WEE1, CDC25C and CDK1 protein and P-Try15-CDK1 phosphorylation levels in nuclear and cytosolic fractions of TRIP12-depleted and control HelaS3 cells after a Ro-3306 treatment were determined by Western blot analysis. 4EBP1 and panH3 protein levels were used as loading and purity controls. Images were obtained from the same experiment and representative of three different experiments. The asterisk and the arrow indicate the position of phosphorylated and non-phosphorylated forms of CDK1, respectively. (**B**) CYCLIN A level in nuclear and cytosolic fractions of TRIP12-depleted and control HelaS3 cells after Ro-3306 treatment was determined by Western blot analysis. 4EBP1 and panH3 protein levels were used as loading and purity controls. Images were obtained from the same experiment and representative of three different experiments. (**C**) CYCLIN A nuclear presence in TRIP12-depleted and control cells after RO-3306 treatment was determined by immunofluorescence. The image illustrates representative cells with nuclear CYCLIN A (white arrows) or not. The graph represents the percentage of cells expressed as mean ± SEM obtained from three different experiments. Percentage of cells in G_2_ phase (white bars), late S (light grey bars) and early S (dark grey bars) was determined by immunofluorescence using criteria defined in Fig. 2C. The bars represent the mean obtained from three different experiments. * and ** indicate a p value < 0.05 and 0.01, respectively. (**D**) Percentage of EDU positive cells in TRIP12-depleted (ShTRIP12) and control (ShScr) HelaS3 cells arrested in early S phase by a double thymidine block treatment and released in the cell cycle was determined by EDU incorporation at the indicated time. The graph represents the mean ± SEM obtained from a minimum of 2500 cells for each indicated time of three different experiments. *, ** and *** indicate a p value < 0.05, 0.01 and 0.001, respectively. (**E**) Percentage of early S and late S cells in EDU-positive TRIP12-depleted (ShTRIP12) and control (ShScr) HelaS3 cells was determined by immunofluorescence using criteria defined in Fig. 2C. The graph represents the mean ± SEM obtained from a minimum of 500 EDU-positive cells of three different experiments. * and ** indicate a p value < 0.05 and 0.01, respectively.

We next aimed to determine the causes of the accelerated mitotic entry of TRIP12-depleted cells. Mitotic entry is governed by a succession of events that leads to the condensation and the segregation of chromosomes into daughter cells. Among them, the Tyr15-dephosphorylation of CDK1 and the kinase WEE1-cytosolic translocation are essential^29,30^. In Ro-3306 cells, we simultaneously found a decreased level of phosphorylation on CDK1-Tyr15 in the nuclear fraction of TRIP12-depleted cells and a WEE1 translocation from the nuclear to the cytosolic fraction **(**Figs. 6A and S5B) which provokes the accelerated entry in prophase when the Ro-3306 is removed (Fig. S5C). It is reported that the cytosolic translocation of WEE1 is mediated by CYCLIN A nuclear import which begins early during S phase to allow DNA replication *via* the activation of the kinase CDK2^31,32^. We measured an increase in CYCLIN A expression in the nuclear fraction that is associated to a decreased expression in cytosolic fraction of these cells (Figs. 6B and S5D). This corroborates with a significant increased proportion of cells with CYCLIN A nuclear staining in TRIP12-depleted cells **(**Fig. 6C**)**. The important role of CYCLIN A in DNA replication led us to investigate the status of DNA replication in CYCLIN A nuclear-positive cells. Our analyses revealed a higher percentage of TRIP12-depleted cells with achieved DNA replication (G_2_-phase cells) and therefore set for mitotic entry (Fig. 6C**)**. More importantly, these results strongly suggest that a TRIP12 depletion accelerates DNA replication. By measuring the duration of S phase, we verified that TRIP12-depleted cells have shortened S phase as compared to controls **(**Fig. 6D**)**. Euchromatin regions are replicated early during S phase when heterochromatin regions are replicated in late S phase. Interestingly, we measured in EDU-positive asynchronous cells a higher proportion of cells in late S phase in TRIP12-depleted cells demonstrating that the depletion of TRIP12 specifically accelerates the DNA replication of euchromatin regions. This result is in accordance with the fact that TRIP12 preferentially colocalizes with euchromatin regions **(**Fig. 2B,C**)**.

### A TRIP12 depletion prolongs the SAC activation, leads to chromosome segregation defects and cell growth inhibition

**Figure 7:**
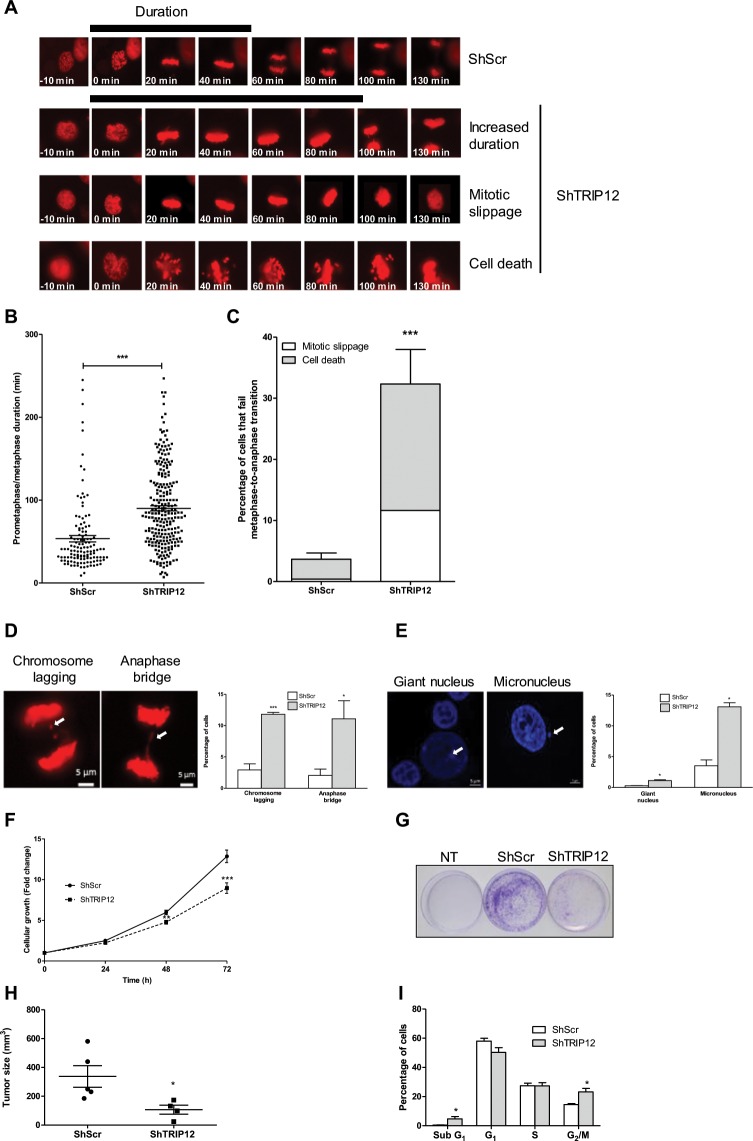
A TRIP12 depletion prolongs the SAC activation, leads to chromosome segregation defects and cell growth inhibition. (**A**) Representative images of the different fate of TRIP12-depleted cells during 140 min. TRIP12-depleted (ShTRIP12#1) and control (ShScr) H2B-dsRed HelaS3 were arrested by a Ro-3306 treatment. Cells were released in the cell cycle and followed by live microscopy for 6 h. The black bar represents the duration of prometaphase-metaphase. (**B**) Duration of prometaphase-metaphase was measured from the invagination of the nuclear membrane to the separation of sister chromatids in 125 control cells and 244 TRIP12#1-depleted cells. The horizontal bar represents the mean ± SEM from three different experiments. *** represents a p value < 0.001. (**C**) Percentage of cells that fail the transition metaphase-to-anaphase in TRIP12#1-depleted and control H2B-dsRed HelaS3 cells. The bars represent the mean ± SEM obtained from three different experiments. *** represents a p value < 0.001. Percentage of cells that exit mitosis *via* mitotic slippage (white bars) and cell death (grey bars). The bars represent the mean obtained from three different experiments. (**D**) Chromosome laggings and anaphase bridges in TRIP12-depleted and control H2B-dsRed HelaS3 cells arrested by Ro-3306 treatment and released in the cell cycle were quantified by live cell microscopy. The images are representative of a chromosome lagging and an anaphase bridge observed in TRIP12-depleted H2B-dsRed HelaS3 cells. The graph represents the percentage of cells with chromosome segregation defects expressed as a mean ± SEM and obtained from 344 TRIP12-depleted and 251 control cells of three different experiments. * and *** indicate a p value < 0.05 and 0.001, respectively. (**E**) Micronucleus and giant nuclei in asynchronous TRIP12-depleted or control HelaS3 cells were quantified by immunofluorescence after DAPI staining. The graph represents the percentage of cells expressed as a mean ± SEM and obtained from a minimum of 10^3^ cells of three different experiments. * indicates a p value < 0.05. (**F**) Cell growth of TRIP12-depleted and control cells was measured by cell counting. Cells were seeded at 5.10^4^ cells/well of 6-wells plate and counted the day after (t = 0) and every 24 h the three following days. Results are expressed as fold change ± SEM relatively to t = 0. Results were obtained from 7 different experiments. ** and *** indicate a p value < 0.01 and 0.001, respectively. (**G**) Colony formation assay of HelaS3 cells transduced or not (NT) with ShScr and ShTRIP12 expressing lentiviral particles. Forty-eight hours after transduction, cells were seeded in a 10 cm-plate and selected with puromycin for 11 days. Colonies were fixed and counterstained with crystal violet. The images are representative of at least three different experiments. (**H**) I*n vivo* tumor progression of TRIP12-depleted and control HelaS3 cells. Cells (10^6^ cells) were sub-cutaneously injected into Scid mice (n = 5). After 5 weeks, mice were sacrificed and tumor volume was determined. Results are expressed in mm^3^ ± SEM. * indicates a p value < 0.05. (**I**) Distribution of TRIP12-depleted and control cells in the cell cycle was determined by flow cytometry after propidium iodide incorporation. The percentage of cells in the different phases of the cell cycle is expressed as a mean ± SEM and was obtained from at least 7 different experiments. * indicates a p value < 0.05.

In addition to accelerate DNA replication, we observed that a TRIP12 depletion can lead to other cellular alterations such as a prolonged activation of the SAC. We observed that in control cells, the percentage of pHH3-Ser10 positive cells reaches a maximum at = 60 min before the physiological loss of pHH3-Ser10 at the metaphase-to-anaphase transition **(**Fig. 5E**)**. In contrast, this percentage persists at a maximal level for 60 min (t = 30 min to t = 90 min) in TRIP12-depleted cells suggesting an inhibition of the metaphase/anaphase transition. This inhibition could be due to a prolonged activation of the SAC or a default of the APC/C complex. The use of reversine, a SAC inhibitor, proved a prolonged activation of the SAC in absence of TRIP12 (Fig. S5E). We confirmed the extended duration of prometaphase-metaphase by measuring a significantly increased duration of prometaphase (from invagination of the nuclear membrane) to metaphase in TRIP12-depleted cells (90.1 min) compared to control cells (53.5 min) **(**Fig. 7A,B**)**. Additionally, we observed that TRIP12-depleted cells largely fail to transit from metaphase to anaphase (32.3% *vs* 3.7%). Among them, 36% exit mitosis by mitotic slippage and 64% die **(**Fig. 7C**)**. Cell death and mitotic slippage are two processes by which cells overcome an extended arrest in prometaphase. A long term arrest in prometaphase and cohesion fatigue can lead to different chromosome segregation abnormalities such as the formation of chromosome laggings, anaphase bridges and alterations that are visible in interphasic cells such as micronuclei or giant nuclei (consequence of mitotic slippage). Among cells that achieve the metaphase-to-anaphase transition, our analysis revealed a higher percentage of chromosome lagging, anaphase bridges, micronuclei and giant nuclei in ShTRIP12 cells (Figs. 7D–E and S5F). These results indicate that TRIP12 also ensures the maintenance of chromosome integrity and therefore the stability of the genome.

The consequences of TRIP12 depletion on S phase duration, mitotic entry and progression led us to investigate the effects on cellular growth. Interestingly, a depletion of TRIP12 provokes a significant diminution of cell growth (30.3% after 72 h) *in vitro* **(**Fig. 7F**)** also visible by colony formation assay **(**Fig. 7G**)** and *in vivo* in SCID mouse model after sub-cutaneous injections **(**Fig. 7H**)**. This reduced cell growth was associated to an accumulation of cells in G_2_/M phase **(**Fig. 7I**)** that could be explained by the combination of the increased duration of prometaphase-metaphase **(**Fig. 7B**)** and the higher percentage of cells that exit mitosis by mitotic slippage with 4n DNA content **(**Fig. 7C**)**. Moreover, this reduced growth is accompanied by an increased proportion of sub-G_1_ cells **(**Fig. 7I**)** which could correspond to the TRIP12-depleted cells that die at the metaphase-to-anaphase transition **(**Fig. 7C**)**.

## Discussion

The E3 ubiquitin ligase TRIP12 was linked to the control of cell cycle regulators (*i.e*.: p14/ARF, p16/CDKN2A)^10^. In this study, we provide evidence that TRIP12 expression varies during the cell cycle and correlates with its nuclear localization. More importantly, we identify a functional N-terminal domain that confers to TRIP12 the capacity to strongly associate with euchromatin. We demonstrate the requirement of TRIP12 in mitotic entry by affecting the duration of S phase. Moreover, we reveal the implication of TRIP12 in the control of SAC activation, proper chromosome segregation and in cell proliferation.

In line with the implication in mitotic entry, TRIP12 over-expression and depletion alter initiation of mitosis. Therefore, fine-tuning of TRIP12 expression and/or activity is essential for the control of this phase. Similar observations are reported for other important mitotic proteins such as CYCLIN A. Indeed, CYCLIN A over-expression delays chromosome alignment and sister chromatid segregation^33^. The knock down of CYCLIN A by siRNA strategy delays the entry into mitosis by postponing cells in G_2_ phase^34^. Although the mechanisms that govern its expression during the cell cycle need to be further studied, our findings indicate that *de novo Trip12* mRNA expression is not involved. These observations corroborate the *Whitfield et al*. study, which provides an exhausting list of HelaS3 mRNAs that fluctuate during cell cycle^35^. *Trip12* mRNA is not present in this list. It is plausible that induction of TRIP12 protein expression in S phase could be mediated by an IRES (Internal Ribosome Entry site) dependent-translational regulation as demonstrated for *Aurora A kinase* mRNA^36^. Comparative proteomics approaches during cell cycle identified TRIP12 as a newly synthetized protein in S phase which is in favor of a translational regulation of *TRIP12* mRNA during cell cycle^37^. Moreover, the disappearance of TRIP12 from the nucleus in G_1_ phase-cells could imply its degradation by the proteasome mediated by APC/C-dependent ubiquitination as TRIP12 possesses putative KEN box (aa 1496–1570/UniProt source) and Destruction boxes (aa 859–867 and aa 1546–1554/ELM resource). This degradation mechanism may explain the decrease of TRIP12 expression. However, other actors that ensure TRIP12 stability remain to be identified. Similar to UHRF1, TRIP12 is stabilized by USP7^12^. During mitosis, CDK1 initiates UHRF1 degradation by phosphorylating the USP7-interacting domain^38^. The same mechanism could exist for TRIP12 degradation since a recent quantitative phospho-proteome approach identified serine 424 residue of TRIP12 as a CDK1 phosphorylation target^39^. Moreover, our preliminary results are in favor of a role of USP7 in the cell cycle regulation of TRIP12. Therefore, the variation of TRIP12 expression during cell cycle likely involves different layers of regulation including translation efficiency and protein stabilization.

Another pressing question is whether TRIP12 exerts its function in mitotic entry through its catalytic activity or by protein-protein interaction. Our observations favor a catalytically-independent function **(**Figs. 4A,B and S4A). However, these experiments were performed in the context of over-expressing the catalytic mutant. In this context, we show that an over-expression of several different TRIP12 domains (except the N-terminal domain) leads to an inhibition of cell division (Fig. S4A–E**)**. In these conditions, we cannot entirely be certain that TRIP12 function in mitotic entry is independent of its catalytic activity. Moreover, TRIP12-GFP transfection leads to uncontrolled TRIP12 expression throughout all cell cycle phases whereas endogenous TRIP12 is tightly regulated during cell cycle. Formulation of a specific inhibitor of TRIP12 enzymatic activity would be required to definitely address this question.

We further demonstrate that TRIP12 interacts with chromatin *via* an IDR. IDRs are found in a multitude of proteins and participate in protein-, DNA-, or RNA-protein interactions^40,41^. DNA binding proteins are significantly enriched in disordered domains in Eukaryotes^42^. Many IDRs are functional, adopting a well-defined conformation upon interaction with target molecules. IDRs constitute important regulatory regions such as the N-terminal part of histones that are subject to numerous post-translational modifications to control the chromatin compaction^41^. To our knowledge, TRIP12 is the first demonstrated E3 ubiquitin ligase interacting with chromatin through an IDR domain. The staining on full-length chromosomes demonstrates that TRIP12 interacts through its IDR with the entire genome suggesting a widespread role of TRIP12 on genome organization and potentially on global gene expression.

We showed that TRIP12 depletion accelerates mitotic entry by shortening the DNA replication phase. DNA replication is a highly ordered process that ensures cells replicate their genome. It requires the sequential assembly of protein complexes such as the pre-replication complex, the pre-initiation complex and the replisome. TRIP12 could participate in the regulation of DNA replication by interacting or controlling the expression of replication complex components. Interestingly, TRIP12 was found in the RPA interactome^43^. Our personal comparative proteomic analysis (SWATH-MS) identified an increased expression of the cyclin dependent kinase CDK6 in TRIP12 depleted cells (Fig. S6A–C**)**. It is known that CDK6 activity is a critical determinant of pre-RC assembly^44^. Therefore, TRIP12 could control DNA replication initiation by regulating CDK6 level. The progression through the different phases of DNA replication can also be controlled by chromatin structures^45^. Our data showed that TRIP12 IDR has the capacity to modify the chromatin structure when expressed at a high level (Fig. S3F,G). It is also reported that TRIP12 associates with chromatin remodeling complex components^4,46,47^. By modifying the structure of chromatin, TRIP12 could regulate S phase progression. In summary, it is likely that TRIP12 controls the duration of DNA replication by multiple complex mechanisms. Their complete discovery will require further investigations.

Our results strongly suggest that in addition to its role for the entry into mitosis, TRIP12 exerts other functions during the progression of mitosis. The interaction of TRIP12 on chromosomes could participate in regulating the kinetics of chromosome condensation or act as a competitor to other chromatin-interacting proteins. The identification of TRIP12 as a potential substrate of CDK1 could also support its role in chromosome condensation. Indeed, it is known that CDK1 substrates, such as CAP-D3 (Chromosome associated protein-D3), are implicated in this process^39,48^. Moreover, histone ubiquitination varies during cell cycle progression. Both histones H2A and H2B are ubiquitinated in S and G_2_ phases, deubiquitinated in prophase and then reubiquinated in anaphase^49^. Also, the promoter of certain active genes remains ubiquitinated during the entire mitosis to facilitate their transcriptional reactivation in post-mitosis^50^. Although the ubiquitin ligase RNF20 is associated to genome ubiquitination, potentially TRIP12 also participates in this epigenetic modification of histones during mitosis.

Finally, our findings show that TRIP12 is localized and sequestered in the nucleus for a short duration. This strict temporal control may act to limit the period during which TRIP12 can exert its nuclear functions, precisely from S to early G_1_. This is of particular importance for the TRIP12 inhibitory function on the DDR by promoting RNF168 degradation^6^. DDR is repressed during mitosis^51^ and reactivated in G_1_ phase to repair DNA damages that pass the G_2_/M checkpoint or created during mitosis. Given its inhibitory role on DDR and nuclear localization during the cell cycle, TRIP12 could participate in the mechanisms of DDR inhibition during mitosis by interacting with chromatin and therefore by preventing chromatin accessibility to RNF168. In contrast, TRIP12 disappearance of the nucleus in G_1_ phase could be part of DDR reactivation in the cell cycle.

In this study, we provide important findings on TRIP12 regulation and its implication in cell cycle progression. More importantly, we reveal for the first time its ability to interact with specific regions of the genome which can prefigure a critical role in the organization and the expression of the genome.

## Materials and Methods

### Cell culture and treatment

HelaS3 cells, HEK-293FT, HelaS3 H2B-dsRed were grown in DMEM 4.5 g/L glucose medium supplemented with 10% fetal calf serum (FCS), L-glutamine and antibiotics (Life Technologies) at 37 °C in humid atmosphere with 5% CO_2_. HelaS3 and HEK-293FT were obtained from the American Type Culture Collection. The HelaS3 H2B-dsRed cell line was generated in the laboratory (see below). For serum starvation, HelaS3 cells were seeded at 80% of confluency and cultured in DMEM 4.5 g/L glucose medium supplemented with 0.5% FCS for 72 h. For double thymidine block, HelaS3 cells were grown in the presence of 2 mM thymidine (Sigma-Aldrich) for 18 h, then in fresh medium for 8 h and further grown with thymidine for another 18 h. Treatments are as follows: HelaS3 cells were cultured in the presence of 2 mM thymidine for 18 h, then in fresh medium for 5 h and treated with 100 ng/mL nocodazole (Sigma-Aldrich) for 5 h. HelaS3 cells were treated with 9 µM Ro-3306 (Tocris Bioscience) for 20 h; or, cells were treated with 500 nM reversine (Sigma-Aldrich) for 2 h. HeLaS3 cells were treated with 10 µM EDU for 15 min. The EDU incorporation was visualized using the Click-It^TM^ EdU Alexa Fluor^TM^ 647 Imaging kit (Life Technologies). The human pancreatic hPNE hTERT cell line was obtained from Dr M. Ouellette (University of Nebraska Medical Center, NE) and grown in DMEM 75% (Life Technologies)/M3 25% (InCell Biotech) medium supplemented with 10% FCS, L-glutamine and antibiotics (Life Technologies), 15 ng/ml EGF (Sigma-Aldrich) and 750 mg/ml puromycin. The human pancreatic cancer cell lines BxPC-3 and Capan-2 were obtained from the American Type Culture Collection and grown in RPMI medium supplemented with 10% FCS, L-glutamine and antibiotics (Life Technologies) at 37 °C in humid atmosphere with 5% CO_2_.

### Plasmids and transfection

The following lentivirus shRNA lentiviral plasmids were purchased from Sigma-Aldrich: pLKO1-TRC1 non mammalian shRNA control SHC002-target sequence CAACAAGATGAAGAGCACCAA, ShTRIP12#1 pLKO1-TRC1 TRCN0000022374-target sequence CCTGAGTCAAGGAAACATGTT, ShTRIP12#2 pLKO1-TRC1 TRCN0000022375-target sequence CCGGAGTTTGAATCCACCTTT and ShTRIP12#3 pLKO1-TRC1 TRCN0000273210-target sequence CCACTACTCAGTCACCTAAAT. HelaS3 cells stably over-expressing histone H2B-dsRed fusion protein were generated after transient transfection of pcDNA_3_/H2B-dsRed plasmid (kind gift from V. Lobjois, Advanced Technology Institute in Life Sciences of Toulouse, France) and selection with hygromycin (800 µg/mL). pENTA0045 plasmid containing TRIP12 cDNA (KIAA0045) was obtained from the Kasuka DNA Research Institute (Japan)^52^. TRIP12-GFP expressing vector was obtained by transferring TRIP12 cDNA into pCDNA^TM^ 6.2/C-EmGFP-DEST vector (Life Technologies). TRIP12-GFP deletion constructs were generated by PCR amplification (Primers are listed in Supplemental Materials and Methods Table 1) followed by insertion into pDONR-201 or pDONR-221 (Life Technologies) using Gateway strategy. The different fragments were subsequently inserted in frame into pCDNA^TM^6.2/C-EmGFP-DEST vector (Life Technologies) using the same strategy. Catalytically inactive TRIP12-GFP C1959A mutant was generated using Quik Change XL Site-Directed mutagenesis kit (Stratagene) and primers listed in Supplemental Materials and Methods Table 1. All plasmid sequences were verified by automatic sequencing. Chloramphenicol Acetyl Transferase (CAT)-GFP expressing vector pcDNA^TM^6.2/C-EmGFP/GW/CAT was purchased from Life Technologies and used as control. Histone H2B (H2B)-GFP expressing plasmid was a gift from D Llères (Institute of Molecular Genetics of Montpellier, France). pSG5-HAx2-FLAG-TRIP12 plasmid was generated by PCR amplification (Primers are listed in Supplemental Materials and Methods Table 1) followed by insertion into the *Kpn* I and *EcoR* V sites of pcDNA_3_ plasmid containing two HA tags, a TEV protease cleavage site and a FLAG motif upstream of the *Kpn* I site. TRIP12 cDNA fragment (1-325) and (446-1000) cloned in pDONR-201 were transferred in pET-60-DEST plasmid (Novagen) by Gateway strategy to generate GST-IDR(1-325) and GST-ARM-WWE(446-1000) plasmids. The different plasmids were transiently transfected in HelaS3 cells using JetPEI™ reagent (PolyPlus-Transfection) following manufacturer’s recommendations with a N/P ratio of 5 or 10.

### Lentiviral vector production and cell transduction

All replication defective, self-inactivating lentiviral vectors were generated in a BSL-3 facility (Vectorology platform, INSERM U1037, Toulouse, France) as previously described by Torrisani *et al*.^53^. Briefly, transient transfection of HEK-293FT cells with packaging and lentiviral vector plasmids were performed using LENTI-Smart INT kit (InvivoGen) following manufacturer’s recommendations. All batches were verified replicative virus-free. Lentiviral vector concentrations were quantified by p24 ELISA (Innotest, Ingen, Paris). Cells were seeded at a density of 10^4^ cells per well in a 48 well-dish. After 24 h, cells were incubated with 150 ng of p24-equivalent of lentiviral vectors in the presence of protamine sulfate (4 µg/mL) for 12 h. Transduced cells were selected for 3 weeks using puromycin (5 µg/mL-InvivoGen).

### Subcellular fractionation and Western blot analysis

Cell pellets were incubated in 100 µM Tris-HCl (pH 7.5), 1,5 mM MgCl_2_, 5 mM KCl, 5 mM DTT, 0,5% NP-40®, 0,5 mM PMSF and 10 µL/mL protease inhibitor (Sigma-Aldrich). After 10 min-incubation on ice, samples were centrifuged (15 min at 2 000 rpm, 4 °C). Supernatants containing cytosolic proteins were kept. After two washes, pellets were resuspended in RIPA (Radio-ImmunoPrecipitation Assay) buffer (Biotech) and centrifuged (20 min at 12 000 rpm, 4 °C), supernatants containing nuclear proteins were kept. The residual pellets were solubilized in RIPA buffer supplemented with 25 U micrococcal nuclease (Takara) and 1 mM DTT. After 20 min-incubation at 37 °C, the reaction was stopped using EDTA solution (44 mM final concentration). After sonication (2 cycles of 6 s, amplitude 40% with Vibra-Cell^TM^ sonicator), samples were centrifuged (15 min at 12 000 rpm, 4 °C). Supernatants containing chromatin fraction were kept. For total protein, cell pellets were incubated in RIPA buffer supplemented with 10 µL/mL protease inhibitor (Sigma-Aldrich). After 15 min on ice, samples are centrifuged (15 min-12 000 rpm, 4 °C). Protein fractions were denaturated in Laemmli buffer after heating at 95 °C for 5 min. Proteins were separated by SDS-PAGE (Sodium Dodecyl Sulfate Polyacrylamide Gel Electrophoresis) and transferred onto nitrocellulose membrane (Bio-Rad) using TransBlot Turbo (Bio-Rad) apparatus. After membrane saturation and primary/secondary antibody incubation, protein expression was detected using Clarity^TM^ Western ECL Substrate (Bio-Rad) and Chemi-Doc^TM^ XRS^+^ (Bio-Rad) apparatus. Signal intensities were quantified using Image Lab (Bio-Rad) software. Antibodies and dilution used for these experiments are listed in Supplemental Materials and Methods Table 2.

### Flow cytometry

For cell cycle analyses, cells were fixed in 70% ethanol during the exponential growth phase for asynchronous HelaS3 cells or after treatment for enriched HelaS3 cell populations. Fixed cells were treated with RNAse A (10 µg/mL) and propidium iodide (20 µg/mL) (Sigma-Aldrich) for 15 min at 37 °C. TRIP12(1-1992)-GFP and H2B-GFP transfected cells were fixed with ethanol 70% and incubated with a pHH3-Ser10 antibody followed by Alexa Fluor® 555 anti-mouse secondary antibody. Data were acquired using the MACS Quant® VYB cytometer (Miltenyi Biotech) and analyzed with MACS Quant and ModFit software. Antibodies and dilution used for these experiments are listed in Supplemental Materials and Methods Table 2.

### Cell proliferation

TRIP12-depleted and control HelaS3 cells were seeded in triplicate at a concentration of 5.10^4^/well of 6 well-plate. After 24 h (t = 0), cells were counted using a Z1 Coulter® Particle Counter (Beckman Coulter™). Cells were counted after 24 h, 48 h and 72 h.

### *In vivo* experiments

Experimental procedures performed on mice were approved by the ethical committee of INSERM- CREFRE (National Institute of Health and Medical Research-Regional Center for Functional Exploration and Experimental Resources) and authorized by the French Ministry of Research: APAFIS#3600-2015121608386111v3. All experiments were performed in accordance with relevant guidelines and regulations. TRIP12-depleted and control HelaS3 cells (1.10^6^ cells) were subcutaneously injected into SCID mice (n = 5). After 5 weeks, mice were euthanized and tumor volume was determined.

### RNA extraction and RT-qPCR

Total RNA was isolated from HelaS3 cell lines with TRIzol® Reagent (Life Technologies) according to supplier’s instructions. One to five µg of total RNA were reverse transcribed into cDNA using RevertAid H minus Reverse Transcriptase kit (Thermo-Scientific) according to manufacturer’s recommendations. Duplicate RT-qPCR assays were carried out in a StepOnePlus™ Real-Time PCR System (Applied Biosystems) with SsoFast™ EvaGreen® supermix (Bio-Rad) and specific primers (see primers listed in Supplemental Materials and Methods 2). Relative quantity of mRNA was calculated by the comparative threshold cycle (CT) method as 2^–ΔCT^, where ΔCT = CT *Trip12* mRNA – CT *Reference* mRNA. Three mRNAs of reference (*β-Actin, Gapdh and cyclophilin A*) were used for normalization. Primers are listed in Supplemental Materials and Methods Table 1.

### Chromosome spreading

Cells were treated with 9 µM Ro-3306 for 20 h, washed with PBS and released in fresh medium for 45 min at 37 °C, or in medium supplemented with 100 µM nocodazole for 4 h. Mitotic cells were collected by brief treatment with trypsin, rinsed with PBS and swollen in hypotonic medium (10 mM KCl, 15% FCS) at 37 °C for 20 min. Cell preparations were fixed with a freshly made 75% ethanol, 25% glacial acetic acid solution overnight at 4 °C, dropped on glass slides and air-dried. Slides were stained with DAPI or processed for immunofluorescence (see below).

### Naked DNA interaction

GST-IDR(1-325) and GST-WWE-ARM(446-1000) recombinant proteins were purified from BL21 bacteria and quantified. Increasing volumes of purified proteins were incubated with 100 ng of pENTR1A-GFP plasmid (made in laboratory) in Tris-HCl (pH 7.6) 10 mM-EDTA 1 mM buffer for 20 min at room temperature. Reactions were migrated on agarose gel 0.7% and visualized with ethidium bromide.

### Immunofluorescence

Cells were grown on cover slips then subsequently fixed and permeabilized using with IntraStain™ reagent (DAKO) or methanol/0.1% Triton™ X-100. Cover slips were saturated using Protein block™ reagent (DAKO) for 30 min and then incubated with primary antibodies overnight at 4 °C. After several washes, cells were incubated with appropriate secondary Alexa Fluor®-488 anti-mouse, Alexa Fluor®-555 anti-mouse or Alexa Fluor®-555 anti-rabbit antibodies for 2 h at room temperature. Nuclei were counterstained with 1 µM DAPI for 5 min at room temperature. Cover slips were mounted on glass slides using Fluorescent Mounting Reagent/Medium (DAKO). Fluorescence was visualized using LSM 780 or 880 confocal microscope (Zeiss) with a 63x NA 1.4 oil-immersion objective and analyzed using Zen software (Zeiss). Antibodies and dilution used for these experiments are listed in Supplemental Materials and Methods Table 2. Images were quantified wit FIJI software.

### Bioinformatic prediction of intrinsically disordered regions

Detection of intrinsically disordered region in TRIP12 protein sequence (NP_004229.1) was determined using the prediction software IUPred (iupred.enzim.hu)^54^, GlobPlot (http://globplot.embl.de/)^55^ and DisEMBL (dis.embl.de)^56^.

### Live cell microscopy

H2B-dsRed HelaS3 cells were treated with Ro-3306 and released in the cell cycle. Fluorescent images were acquired (Objective 20x) every minute for 6 h using a Zeiss motorized inverted Observer Z1 microscope, containing LED module Colibri. Filter combinations: GFP (38 HE) DsRed (43 HE) and DAPI (49) with the AxioCam MRm camera system. Time-lapse imaging of living cells was performed using Pecon-Zeiss incubation system for temperature (37 °C) and CO_2_ (5%) controlled environment. Images were subsequently analyzed using ZEN SP2 software (Blue edition Zeiss). Transfected Ro-3306-treated HelaS3 cells released in the cell cycle were imaged every half-hour or hour using IncuCyte Zoom Kinetic Imaging System with a 10x objective.

### SWATH-MS comparative proteomic analysis

Analysis of proteins was performed using a microLC system Ultimate 3000 (Dionex, Villebon sur Yvette, France) coupled to a Triple-TOF 5600 + (AB Sciex, Les Ullis, France) in the positive ion mode. Samples were first dissolved in 16 _L of buffer (5% ACN, 0.05% trifluoroacetic acid) and spiked with iRT calibration mix (Biognosys, Schlieren, France). The totality of the samples was then injected on a YMC-Pack Pro C18 column (3.0 mm _ 150 mm; 3 _m particle size) at a flow rate of 5 _L.min^−1^. The run length was over 90 min with a gradient from 7% to 45% buffer B (buffer A: 0.1% formic acid, buffer B: 90% ACN, and 0.1% formic acid) in 70 min. The MS data were acquired with a SWATH mode. The source parameters were set as follows: IS at 5500 V, Cur gas at 25, GS1 at 5. The acquisition parameters were as follows: one 50 msec accumulation time MS scan followed by 50 variable SWATH windows each at 40 msec accumulation time for m/z 400–1250. Identification was determined using an in-house SWATH library created HelaS3 cells with MaxQuant software, Les Ulis, France) (FDR 1%). A mass accuracy of 20ppm on precursor ions was used, and 0.5 Da on the fragments. Cysteine carbamidomethylation, methionine oxidation, proline hydroxylation and serine, threonine and tyrosine phosphorylations were taken into account. Data treatment was done with Spectronaut Software 9.0, Les Ulis, France). Analysis of ShScr and ShTRIP12#1 HelaS3 cells was performed on three different transductions. For each peptide, a triplicate with a peptide quantity <5 and a standard error >60% of the mean was excluded from the analysis. For each replicate, the sum of the four most abundant peptides was calculated. The mean of ShTRIP12 triplicate was compared to the one of ShScr triplicate. Protein with a fold change >1.2 and <0.8 compared to ShScr cells were considered.

### Statistical analysis

*In vitro* data were analyzed by 2-tailed, unpaired Student’s t-test using a multiple statistics Graph Pad Prism 5 software package and a difference was considered significant when p value was lower than 0.05. Mean values are given ± SEM. Number of independent experiments is indicated in the figure legends. *, ** and *** Indicate a p value < 0.05, 0.01 and 0.001, respectively.

## Data availabilty

The datasets generated during and/or analyzed during the current study are available from the corresponding author on reasonable request.

## Supplementary information

Supplementary information.
