# Supplementary material for: The E3 ubiquitin ligase TRIP12 participates in cell cycle progression and chromosome stability
Source: Sci Rep. 2020 Jan 21;10:789. doi: 10.1038/s41598-020-57762-9 (PMC6972862; doi:10.1038/s41598-020-57762-9)
Supplement: Supplementary file 1 — Supplementary information. [file 41598_2020_57762_MOESM1_ESM.pdf]

Supplementary Information

The E3 ubiquitin ligase TRIP12 participates in cell cycle progression and chromosome stability.

Larrieu D[1], Brunet M[1], Vargas C[1], Hanoun N[1], Ligat L[1], Dagnon L[1], Lulka H[1], Pommier RM[2], Selves J[1], Jády BE[3], Bartholin L[2], Cordelier P[1], Dufresne M[1] and Torrisani J.*[1]

Supplemental Figure 1

A

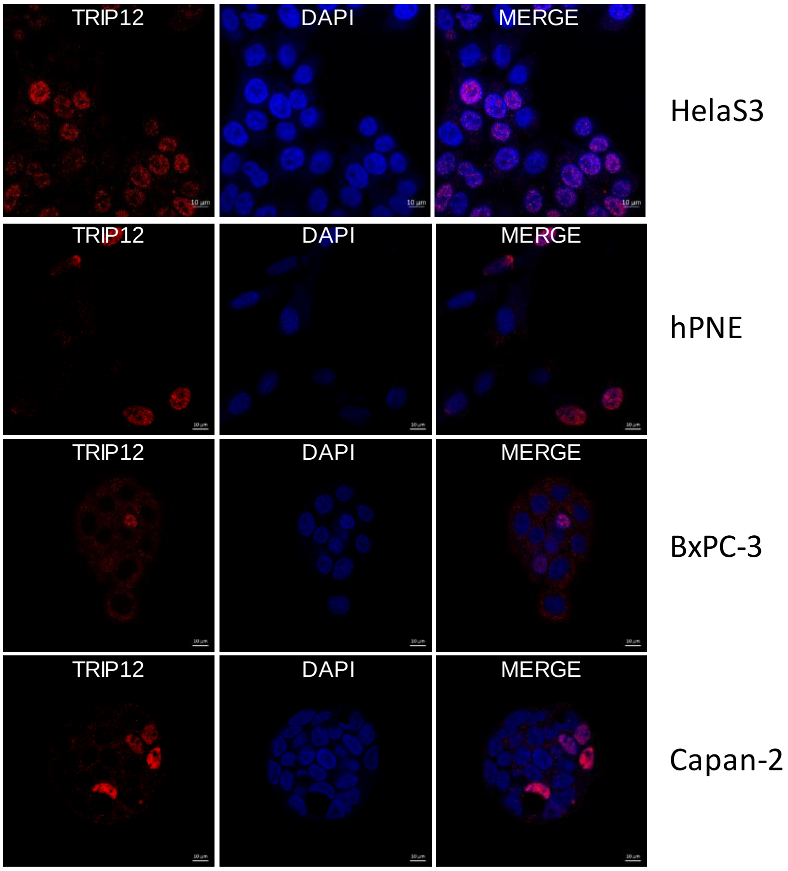

B

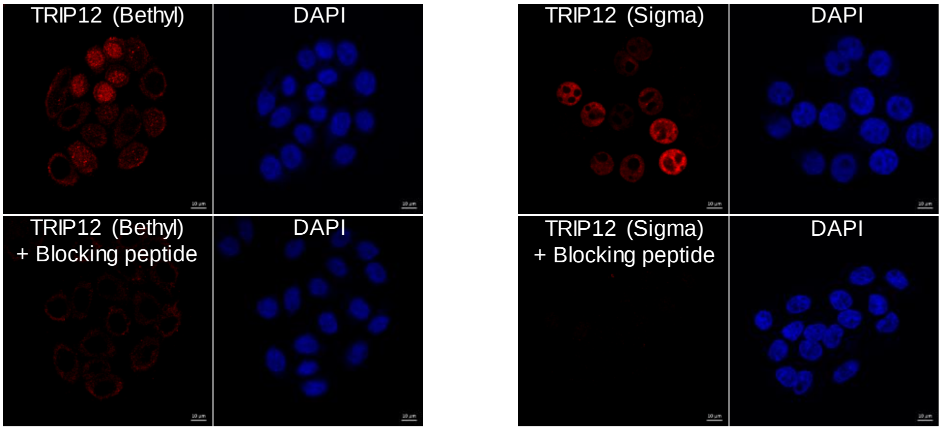

C

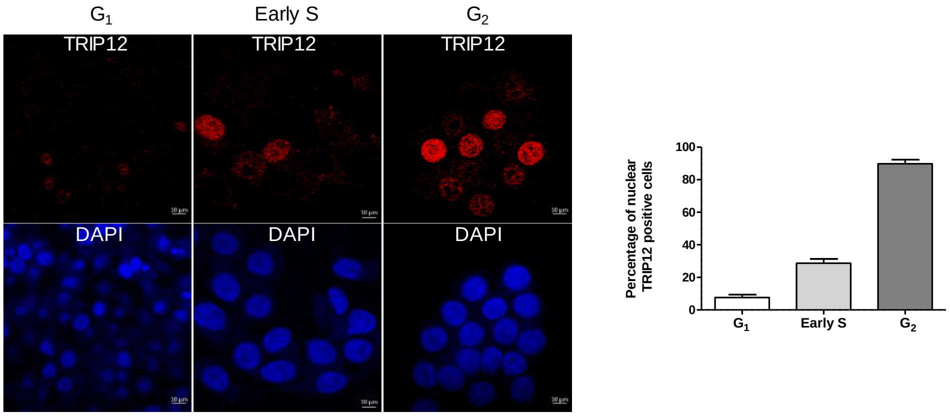

Supplemental Figure 2

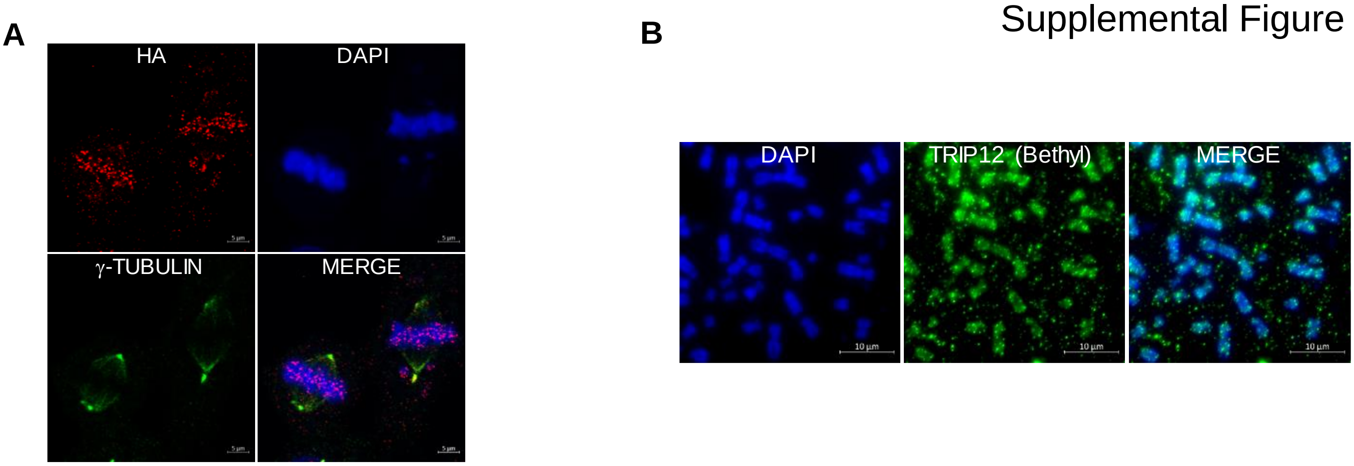

Supplemental Figure 3

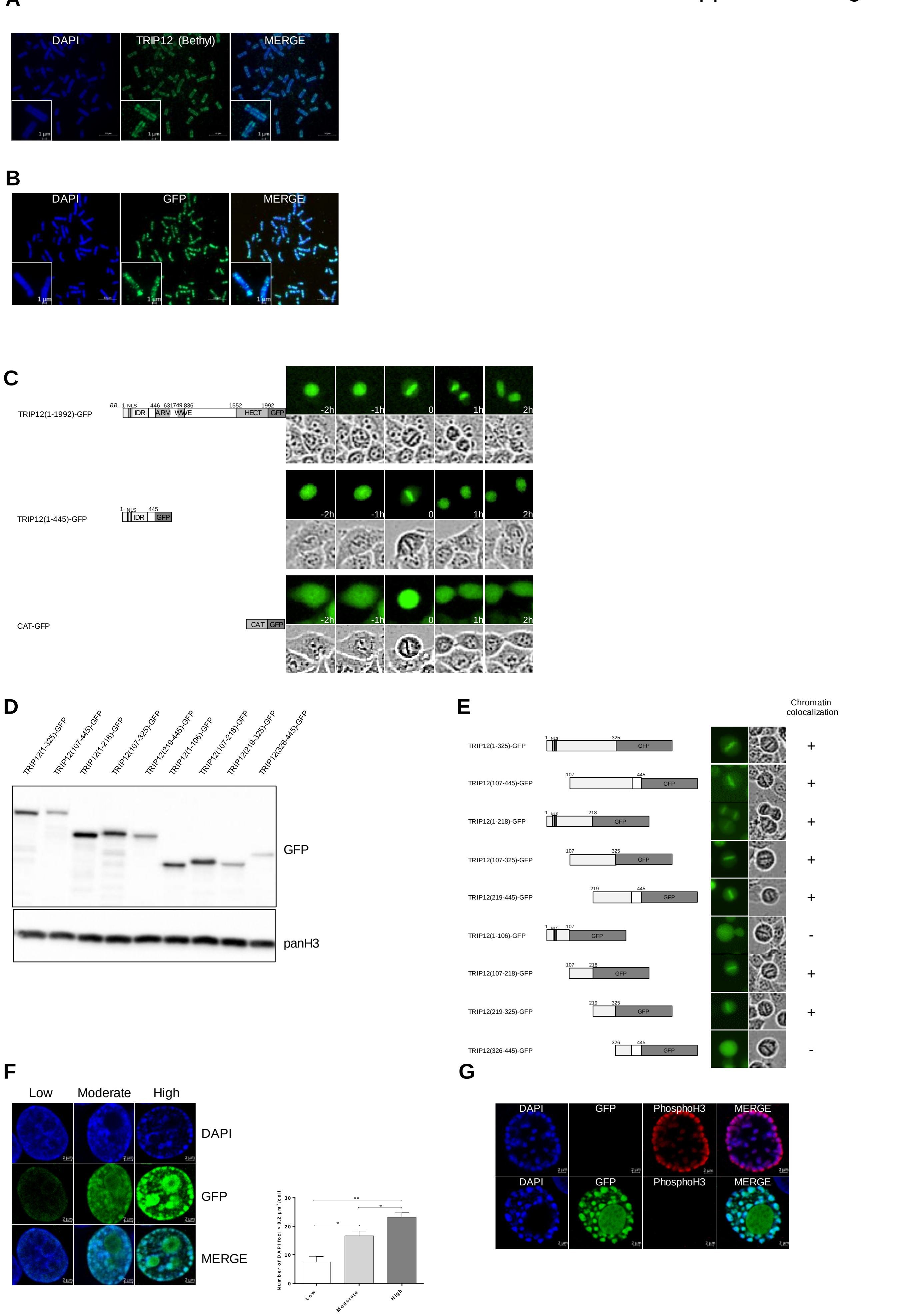

Supplemental Figure 4

A

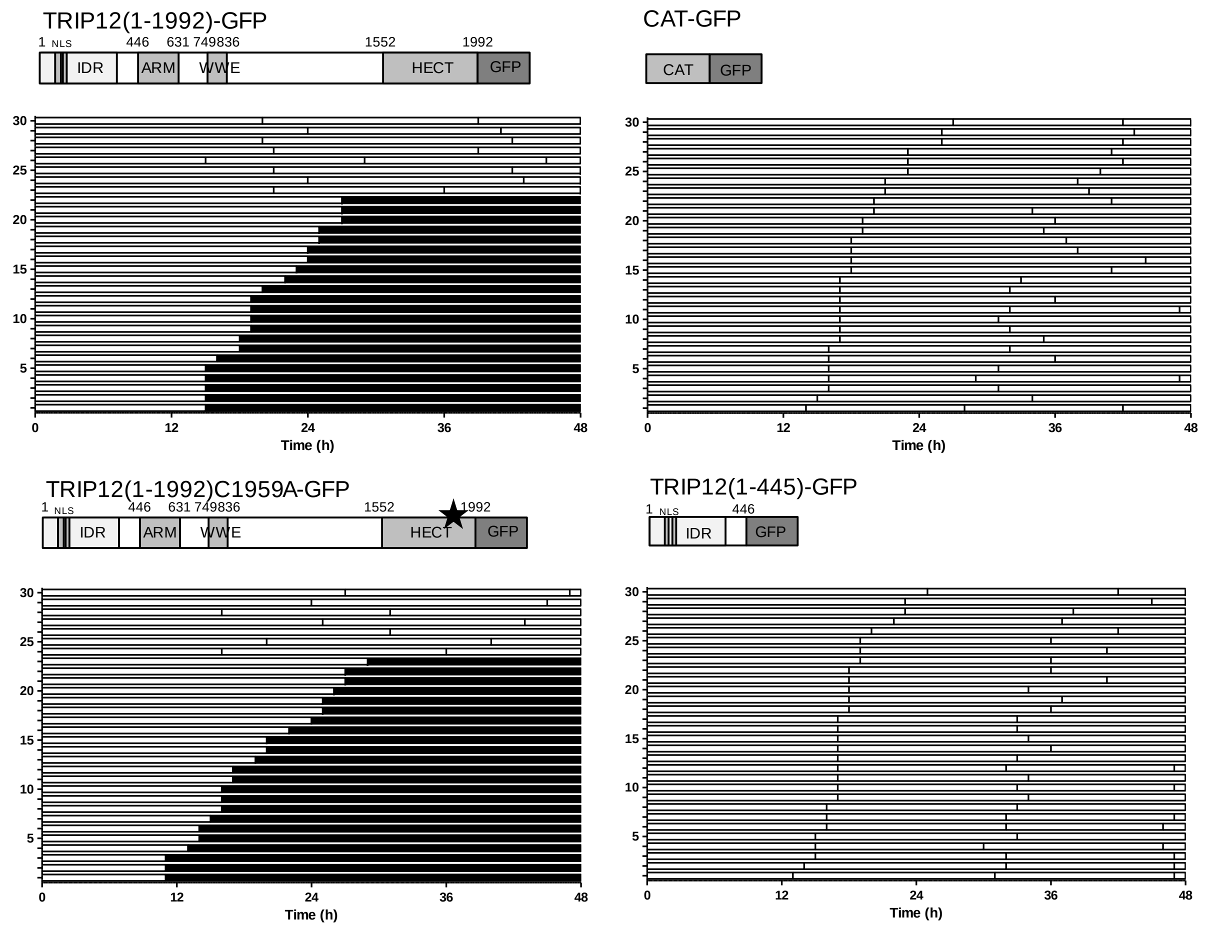

Supplemental Figure 4

B

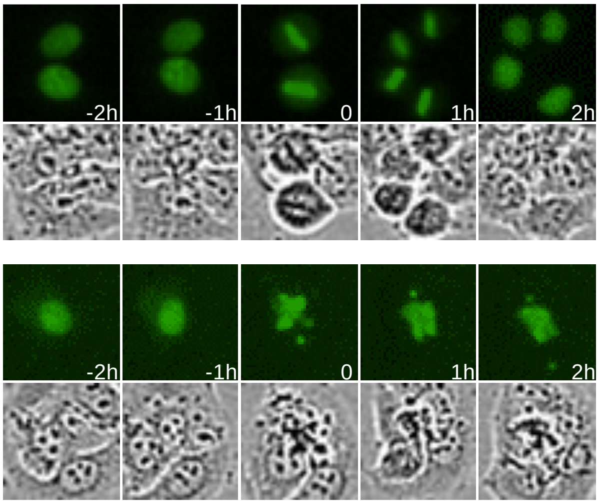

C

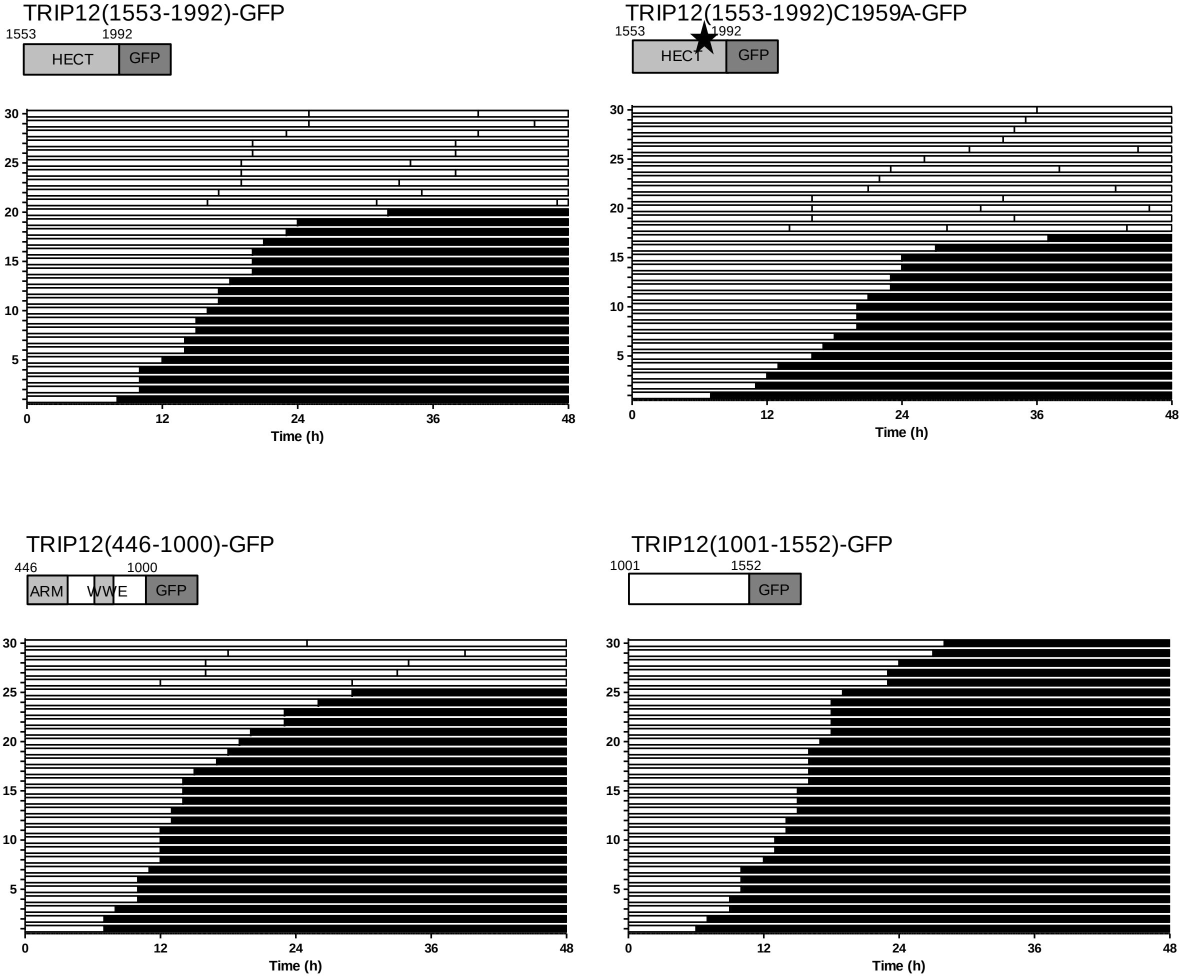

D

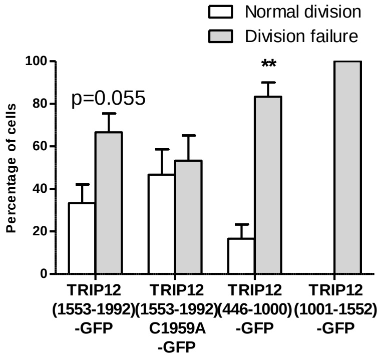

E

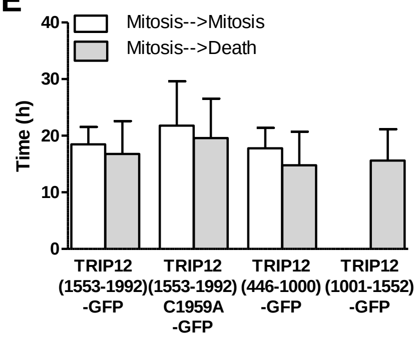

Supplemental Figure 5

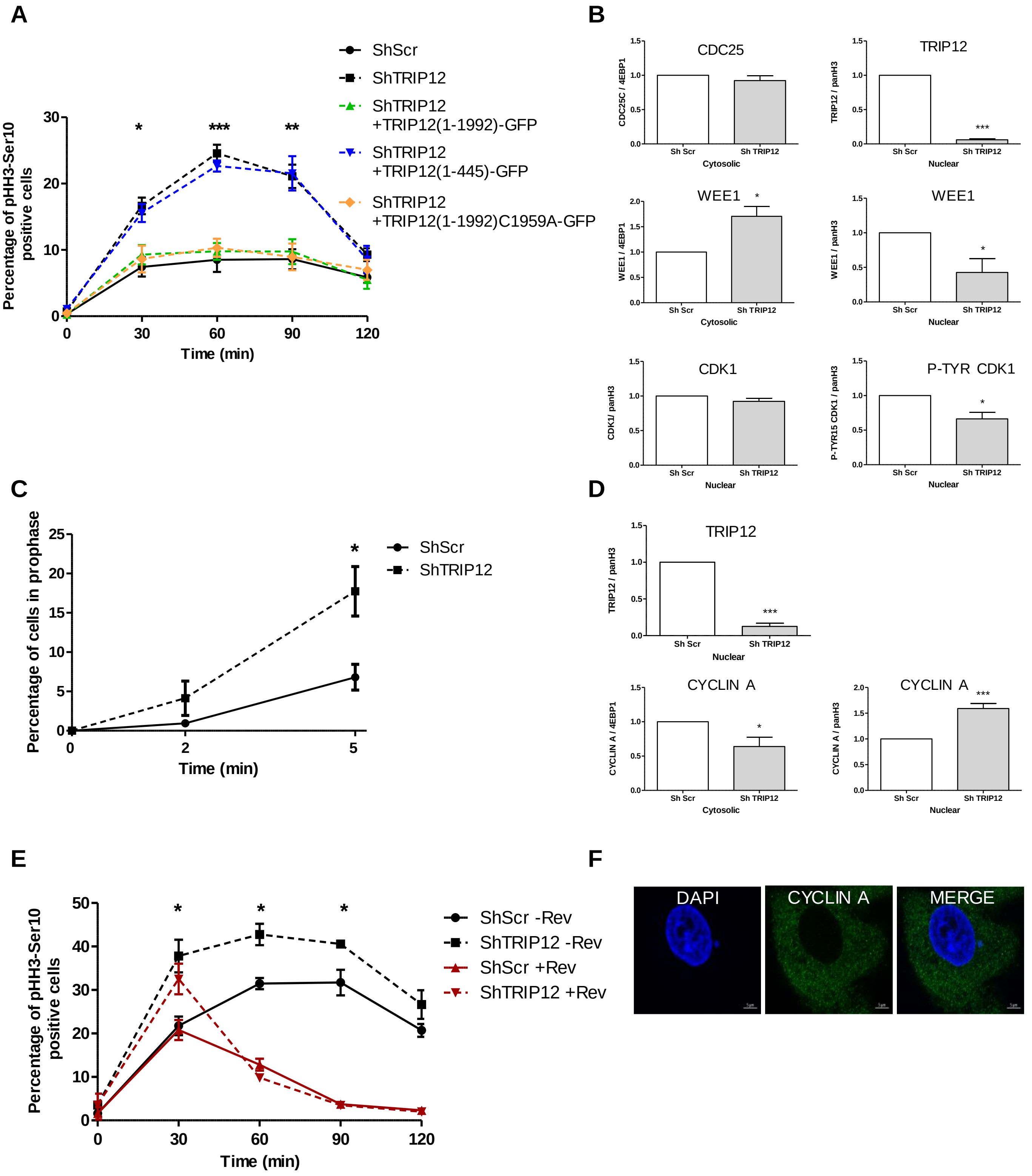

A

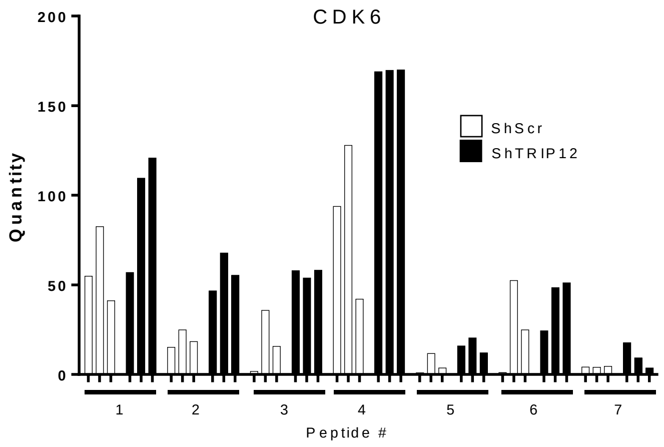

B

Peptide #	EG.PrecursorId	PG.Genes	PG.ProteinDescr	PG.ProteinName	PG.UniProtId	Scr 1 quantity	Scr 2 quantit	Scr 3 quantit	ShTRIP12 1 q	ShTRIP12 2 q	ShTRIP12 3 q
1	_DMMFQLLR_.2	CDK6	Cyclin-dependen	CDK6_HUMAN	Q00534	54.8704756	82.5543936	41.1893863	56.946542	109.485726	120.822676
2	_HLETFEHPNVVR_.3	CDK6	Cyclin-dependen	CDK6_HUMAN	Q00534	15.2014343	24.9667049	18.3457	46.654626	67.8512555	55.3195696
3	_DLKPQNILVTSSGQIK_.3	CDK6	Cyclin-dependen	CDK6_HUMAN	Q00534	1.66624045	35.7667388	15.7377938	57.9798077	53.8774359	58.1437529
4	_ILDVIGLPGEEDWPR_.2	CDK6	Cyclin-dependen	CDK6_HUMAN	Q00534	93.8028713	127.805302	42.029027	168.89645	169.712144	169.922299
5	_GSSDVDQLGK_.2	CDK6	Cyclin-dependen	CDK6_HUMAN	Q00534	1	11.666137	3.70591633	15.956683	20.4608182	12.1432587
6	_ISAYSALSHPYFQDLER_.3	CDK6	Cyclin-dependen	CDK6_HUMAN	Q00534	1.14878367	52.4261531	24.908231	24.3489882	48.4398565	51.1146844
7	_LFDVC[+57]TVSR_.2	CDK6	Cyclin-dependen	CDK6_HUMAN	Q00534	4.12464863	4.0699701	4.52272557	17.7130535	9.23524158	3.62911592

C

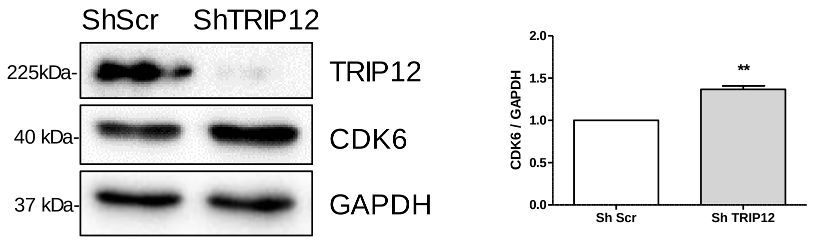

Supplemental Figure Legends

Supplemental Figure 1.

A- TRIP12 localization in HelaS3, hPNE, BxPC-3 and Capan-2 cells was determined by immunofluorescence with anti-TRIP12 antibody (Bethyl). Nuclei were counterstained with DAPI.

B- Specificity of anti-TRIP12 antibodies was assessed by immunofluorescence. HelaS3 cells were immuno-stained using anti-TRIP12 Bethyl and Sigma antibodies in presence or not of corresponding blocking peptide (in 50X excess). Nuclei were counterstained with DAPI. The cytosolic staining with Bethyl antibody corresponds to a non-specific staining.

C- TRIP12 subcellular localization in G_1, early S and G_2-enriched HelaS3 cell populations was determined by immunofluorescence using TRIP12 antibody (Bethyl). Nuclei were counterstained with DAPI. Results are expressed as percentage of cells ± SEM obtained from a minimum of 400 cells in three independent experiments.

Supplemental Figure 2.

A- TRIP12 localization in HelaS3 cells in metaphase transfected with HA-TRIP12 construct was visualized by immunofluorescence using anti-HA antibody. An anti-γ-TUBULIN antibody was used as a spindle marker. Nuclei were counterstained with DAPI.

B- TRIP12 localization on metaphasic chromosomes was visualized by immunofluorescence using anti-TRIP12 antibody (Bethyl) after chromosome spreading of HelaS3 cells treated with Ro-3306 and released in the cell cycle for 45 min. DNA was counterstained with DAPI.

Supplemental Figure 3.

A- TRIP12(1-445)-GFP on metaphasic chromosome arms was visualized by immunofluorescence using anti-TRIP12 antibody (Bethyl) after chromosome spreading of HelaS3 cells transfected with TRIP12(1-445)-GFP construct treated with Ro-3306 and released in the cell cycle for 45 min. DNA was counterstained with DAPI. The inset represents a magnification of TRIP12 localization on an individual chromosome.

B- TRIP12(1-445)-GFP localization on metaphasic chromosomes was visualized by immunofluorescence using anti-GFP antibody after chromosome spreading of HelaS3 cells transfected with TRIP12(1-445)-GFP construct treated with Ro-3306 and released in the cell cycle for 45 min. DNA was counterstained with DAPI.

C- GFP-fusion protein localization in living HelaS3 cells previously transfected with TRIP12(1-1992) and (1-445)-GFP constructs was followed by live cell microscopy. CAT-GFP was used as control. GFP and phase contrast cells visualization were presented 1h and 2h before and after a representative cell in metaphase.

D- Expression of TRIP12-GFP constructs was measured by Western blot 24h after transient transfection in HelaS3 cells. PanH3 protein level was used as loading control. Images are representative of three different experiments.

E- GFP-fusion protein localization in living HelaS3 cells previously transfected with a series of TRIP12-GFP constructs as indicated was followed by live cell microscopy. Visualization of GFP and phase contrast cells was presented in representative cells in metaphase. + and – indicate colocalization of GFP-constructs with chromatin or not.

F- Number of DAPI foci in interphasic HelaS3 cells expressing low, moderate and high concentration of TRIP12(1-445)-GFP construct was visualized by immunofluorescence using an anti-GFP antibody. Nuclei were counterstained with DAPI. Results are expressed as number of DAPI foci > 0.2 μm^2/cell ± SEM obtained from a minimum of three different cells per category. * and ** indicate a p value < 0.05 and 0.01, respectively.

G- GFP and pHH3-Ser10 expression in HelaS3 cell in prophase (top panel) or highly expressing TRIP12(1-445)-GFP construct (bottom panel) was visualized by immunofluorescence. Nuclei were counterstained with DAPI.

Supplemental Figure 4.

A- GFP-positive HelaS3 cells were imaged every hour for 48h after transient transfection with TRIP12(1-1992)-GFP, TRIP12(1-1992-C1959A)-GFP, TRIP12(1-445)-GFP and CAT-GFP constructs. Thirty GFP-positive cells obtained from three different experiments were analyzed. Each white horizontal bar represents time spent in interphase of one cell. Each vertical bar between white bars indicates cell division. Bars in black correspond to time spent after cell death.

B- Illustrative images of TRIP12(1-1992)-GFP transfected cells that overcome cell division (top panel) or die (bottom panel) obtained by live cell microscopy.

C- GFP-positive HelaS3 cells were imaged every hour for 48h after transient transfection with TRIP12(1553-1992)-GFP, TRIP12(1553-1992) C1959A-GFP, TRIP12(446-1000)-GFP, TRIP12(1001-1552)-GFP constructs. Thirty GFP-positive cells obtained from three different experiments were analyzed. Each white horizontal bar represents time spent in interphase of one cell. Each vertical bar between white bars indicates cell division. Bars in black correspond to time spent after cell death.

D- The graph represents the percentage ± SEM of GFP-cells that overcome at least two cell divisions or die over a 48h-period. ** indicates a p value < 0.01.

E- The graph represents the average duration ± SEM in interphase between two mitoses (white bars) and between mitosis and cell death (grey bars) in the different GFP positive cells.

Supplemental Figure 5.

A- Percentage of pHH3-Ser10 positive in TRIP12-depleted (ShTRIP12#1) and control (ShScr) HelaS3 cells arrested by Ro-3306 treatment was assessed by flow cytometry after release in inhibitor free medium every 30 min for 2h. TRIP12-depleted cells were previously transfected with TRIP12(1-1992)-GFP, TRIP12(1-1992) C1959A-GFP or control TRIP12(1-445)-GFP constructs. The graph represents the mean ± SEM of three different experiments. *, **, *** indicate a p value < 0.05, < 0.01 and < 0.001, respectively.

B- Quantification of TRIP12, CDC25, WEE1, P-Tyr15 CDK1 and CDK1 protein expression in cytosolic and nuclear fractions of TRIP12-depleted and control HelaS3 cells was determined by Western blot analysis. The graphs represent the mean ± SEM of three different experiments. * and *** indicate a p value < 0.05 and 0.001, respectively.

C- Percentage of cells in prophase in TRIP12-depleted and control HelaS3 cells arrested by a Ro-3306 treatment and released in the cell cycle was determined by immunofluorescence using anti-pHH3-Ser10 antibody at the indicated time. The results represent the mean ± SEM obtained from at least 10^4 cells of three different experiments. * indicates a p value < 0.05.

D- Quantification of TRIP12 and CYCLIN A protein expression in cytosolic and nuclear fractions of TRIP12-depleted and control HelaS3 cells was determined by Western blot analysis. The graphs represent the mean ± SEM of three different experiments. * and *** indicate a p value < 0.05 and 0.001, respectively.

E- Percentage of pHH3-Ser10 positive cells in TRIP12-depleted (ShTRIP12#1) and control (ShScr) HelaS3 cells arrested in G_2 phase by a Ro-3306 treatment and released in the cell cycle by medium replacement was determined by flow cytometry at the indicated time. Reversine (500 nM) was added in the medium at time 0. The results represent the mean ± SEM obtained from three different experiments. * indicates a p value < 0.05.

F- CYCLIN A localization in HelaS3 cells with micronucleus was visualized by immunofluorescence. Nuclei were counterstained with DAPI.

Supplemental Figure 6.

A- Quantification of 7 peptides corresponding to CDK6 protein in TRIP12-depleted and control HelaS3 cells was determined by SWATH-MS analysis (Supplemental Materials and Methods). Each bar represents a biological replicate.

B- Raw data corresponding to CDK6 peptides identified by SWATH-MS.

C- CDK6 expression in TRIP12-depleted and control HelaS3 cells was determined by Western blot analysis. The GAPDH protein level was used as loading control. Images were obtained from the same experiment and representative of three different experiments. The graph represents the mean expression of CDK6 ± SEM. ** indicates a p value < 0.01.

Table 1: List of primers.

For RT-qPCR.

Trip12 Forward: 5’-TTCAGATTGGTGGACCTTCC-3’; Reverse: 5’-GGCTACAACTGGGTCGATGT-3’

Cyclin B1 Forward: 5’-CAAGCCCAATGGAAACATCTG-3’; Reverse: 5’-TTGCTCTTCCTCAAGTTGTCTC-3’

Gapdh Forward: 5’-CAATGACCCCTTCATTGACC-3’; Reverse: 5’-GTACTCGCTCCTGGAAGATG-3’

β-Actin Forward: 5’-AGATGTGGATCAGCAAGCAGGAGT-3’; Reverse: 5’-GCAATCAAAGTCCTCGGCC
hACATT-3’

Cyclophilin A Forward: 5’-GTCAACCCCACCGTGTTCTT-3’; Reverse: 5’-CTGCTGTCTTTGGGACCTTGT-3’

For deletion constructs.

TRIP12(1-1992)-GFP Forward: 5’-GGGGACAAGTTTGTACAAAAAAGCAGGCTTCGAAGGAGATAGAACCATGTCCAACC
GGCCTAATAAC-3’; Reverse: 5’-GGGGACCACTTTGTACAAGAAAGCTGGGTTGTAGGAAAGATGGAACGACTG-3’

TRIP12(1-1552)-GFP Forward: 5’-GGGGACAAGTTTGTACAAAAAAGCAGGCTTCGAAGGAGATAGAACCATGTCCAACC
GGCCTAATAAC-3’; Reverse: 5’-GGGGACCACTTTGTACAAGAAAGCTGGGTTGTAATAAAAAAGCATTTGCCGGGTATCA
AAAGG-3’

TRIP12(1-445)-GFP Forward: 5’-GGGGACAAGTTTGTACAAAAAAGCAGGCTTCGAAGGAGATAGAACCATGTCCAACC
GGCCTAATAAC-3’; Reverse: 5’-GGGGACCACTTTGTACAAGAAAGCTGGGTTGTATCCTTGTAGTAGCTGCTGGGC-3’

TRIP12(446-1551)-GFP Forward: 5’-GGGGACAAGTTTGTACAAAAAAGCAGGCTTCGAAGGAGATAGAACCATGTTGC
AAGCCAGTGATGAAAGT-3’; Reverse: 5’-GGGGACCACTTTGTACAAGAAAGCTGGGTTGTAATAAAAAAGCATTTGCC
GGGTATCAAAAGG-3’

TRIP12(1553-1992)-GFP Forward: 5’-GGGGACAAGTTTGTACAAAAAAGCAGGCTTCGAAGGAGATAGAACCATGGT
AACTGCATTTGATCGGGAC-3’; Reverse: 5’-GGGGACCACTTTGTACAAGAAAGCTGGGTTGTAGGAAAGATGGAACG
ACTG-3’

TRIP12(446-1000)-GFP Forward: 5’-GGGGACAAGTTTGTACAAAAAAGCAGGCTTCGAAGGAGATAGAACCATGTTGC
AAGCCAGTGATGAAAGT-3’; Reverse: 5’-GGGGACCACTTTGTACAAGAAAGCTGGGTTGTATAGAACATCACTTAATCG-3’

TRIP12(1000-1552)-GFP Forward: 5’-GGGGACAAGTTTGTACAAAAAAGCAGGCTTCGAAGGAGATAGAACCATGAAGAGA
AAACGACTGCCAAAA-3’; Reverse: 5’-GGGGACCACTTTGTACAAGAAAGCTGGGTTGTAATAAAAAAGCATTTGCCGGGTAT
CAAAAGG-3’

TRIP12(1-325)-GFP Forward: 5’-GGGGACAAGTTTGTACAAAAAAGCAGGCTTCGAAGGAGATAGAACCATGTCCAACCGG
CCTAATAAC-3’; Reverse: 5’-GGGGACCACTTTGTACAAGAAAGCTGGGTTGTAGCGTGTGCTCCGCCTC-3’

TRIP12(107-445)-GFP Forward: 5’-GGGGACAAGTTTGTACAAAAAAGCAGGCTTCGAAGGAGATAGAACCATGCCACATA
GTAAGTCAAAGAAGAGAC-3’; Reverse: 5’-GGGGACCACTTTGTACAAGAAAGCTGGGTTGTATCCTTGTAGTAGCTGCTG
GGC-3’

TRIP12(1-218)-GFP Forward: 5’-GGGGACAAGTTTGTACAAAAAAGCAGGCTTCGAAGGAGATAGAACCATGTCCAACCGG
CCTAATAAC-3’; Reverse: 5’-GGGGACCACTTTGTACAAGAAAGCTGGGTTGTATGGTGGTACAGTGGAGGAG-3’

TRIP12(107-325)-GFP Forward: 5’-GGGGACAAGTTTGTACAAAAAAGCAGGCTTCGAAGGAGATAGAACCATGCCACATA
GTAAGTCAAAGAAGAGAC-3’; Reverse: 5’-GGGGACCACTTTGTACAAGAAAGCTGGGTTGTAGCGTGTGCTCCGCCTC-3’

TRIP12(219-445)-GFP Forward: 5’-GGGGACAAGTTTGTACAAAAAAGCAGGCTTCGAAGGAGATAGAACCATGGGTGCC
AGAGTGAAACAAGG-3’; Reverse: 5’-GGGGACCACTTTGTACAAGAAAGCTGGGTTGTATCCTTGTAGTAGCTGCTGGGC-3’

TRIP12(1-106)-GFP Forward: 5’-GGGGACAAGTTTGTACAAAAAAGCAGGCTTCGAAGGAGATAGAACCATGTCCAACCGG
CCTAATAAC-3’; Reverse: -5’- GGGGACCACTTTGTACAAGAAAGCTGGGTTGTACTTATTTGTTTCTGAGGGAGATTCAG-3’

TRIP12(107-218)-GFP Forward: 5’-GGGGACAAGTTTGTACAAAAAAGCAGGCTTCGAAGGAGATAGAACCATGCCACA
TAGTAAGTCAAAGAAGAGAC-3’; Reverse: 5’-GGGGACCACTTTGTACAAGAAAGCTGGGTTGTATGGTGGTACAGTGGA
GGAG-3’

TRIP12(219-325)-GFP Forward: 5’-GGGGACAAGTTTGTACAAAAAAGCAGGCTTCGAAGGAGATAGAACCATGGG
TGCCAGAGTGAAACAAGG-3’; Reverse : 5’-GGGGACCACTTTGTACAAGAAAGCTGGGTTGTAGCGTGTGCTCCGCCTC-3’

TRIP12(326-445)-GFP Forward: 5’-GGGGACAAGTTTGTACAAAAAAGCAGGCTTCGAAGGAGATAGAACCATGCAAAAGA
CCACGGGCTCC; Reverse: 5’-GGGGACCACTTTGTACAAGAAAGCTGGGTTGTATCCTTGTAGTAGCTGCTGGGC-3’

For site-directed mutagenesis.

TRIP12(1-1992)-GFP C1959A Forward: 5’-GCCCTCTGTAATGACTGCTGTGAACTATCTTAAGTTGCCGG-3’; Reverse 5’-CCGGCAACTTAAGATAGTTCACAGCAGTCATTACAGAGGGC-3’

For psg5 HAx2-FLAG-TRIP12 construct Forward: 5’-ATAGGTACCATGTCCAACCGGCCTAATAACAATCC-3’; Reverse: 5’-ATAGATATCTTAGGAAAGATGGAACGACTGCTGCCC-3’

Table 2: List of antibodies and peptides.

Proteins	Primary antibodies	Dilution (type of assay)
TRIP12	Bethyl laboratories A301-814A	1/1000 (IF and WB)
TRIP12	Sigma HPA036835	1/1000 (IF)
CYCLIN B1	Millipore MAB3684	1/1000 (IF and WB)
CYCLIN A	Santa Cruz sc-751	1/1000 (IF)
CYCLIN A	GeneTex GTX634420	1/1000 (WB)
Phospho HISTONE H3 (Ser 10)	Sigma H0412	1/500 (WB)
Phospho HISTONE H3 (Ser 10)	Cell Signaling 9706	1/1000 (IF and FACS)
GAPDH	Santa Cruz sc-25778	1/2000 (WB)
γ-TUBULIN	Abcam Ab27074 [Tu30]	1/500 (IF)
GFP	Santa Cruz sc-9996	1/200 (IF)
4EBP1	Cell Signaling 9644	1/5000 (WB)
SP1	Santa Cruz sc-59	1/500 (WB)
panH3	Upstate 07-690	1/6000 (WB)
WEE1	Cell Signaling 4936	1/1000 (WB)
CDC25C	Santa Cruz 327	1/1000 (WB)
Phospho CDK1 (Tyr15)	Cell Signaling 9111	1/2000 (WB)
CDK1	Cell Signaling 28439	1/1000 (WB)
HA-tag	Cell Signaling 3724	1/500 (IF)
CDK6	Santa Cruz sc-177	1/1000 (WB)

Secondary antibodies:
Alexa-Fluor®555 anti-rabbit and anti-mouse secondary antibodies (Life Technologies). Dilution IF:1/1000.
Alexa-Fluor®488 anti-mouse secondary antibody (Life Technologies). Dilution IF: 1/1000 and dilution FACS: 1/200.
Peptides:
Recombinant protein fragment of human TRIP12 (PrEST Antigen TRIP12 #APrEST79147).
TRIP12 blocking peptide (Bethyl Laboratories #BP301-814).

Original Western blot membranes.

Fig. 1E

Exposure time 1sec for TRIP12:

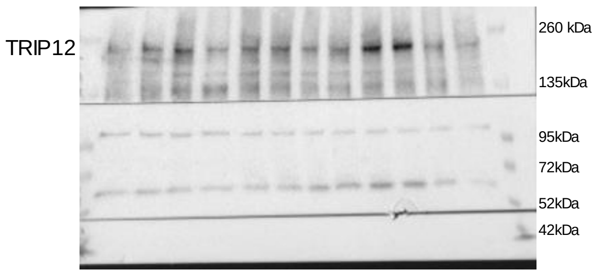

Exposure time 12,3 sec for CYCLIN B1:

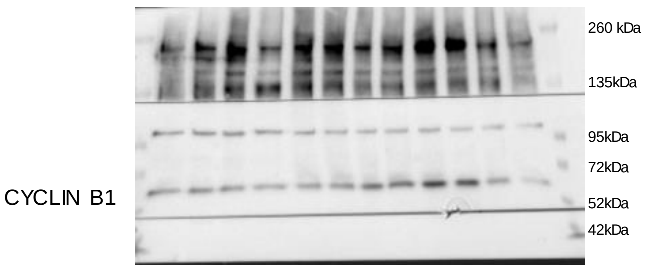

Exposure time 3,3 sec for pHH3-Ser10:

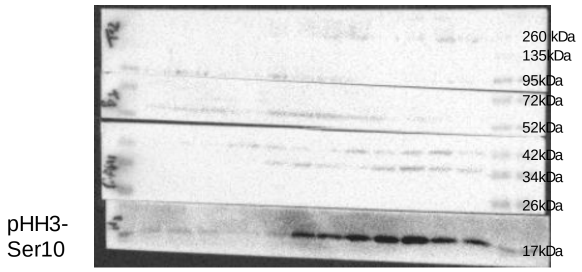

Exposure time 1 sec for GAPDH:

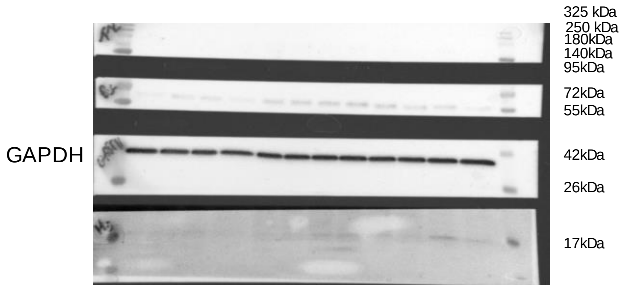

Fig. 1F

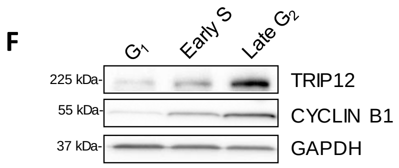

Exposure time 1sec for TRIP12:

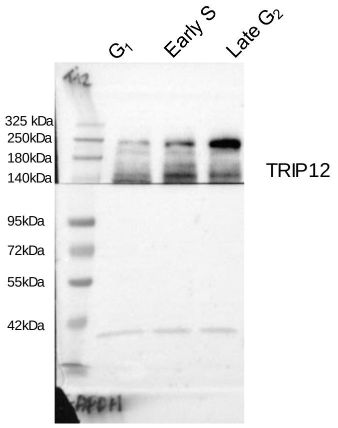

Exposure time 28sec for GAPDH:

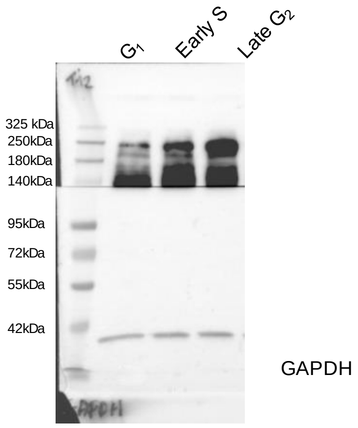

Exposure time 16,2sec for CYCLIN B1:

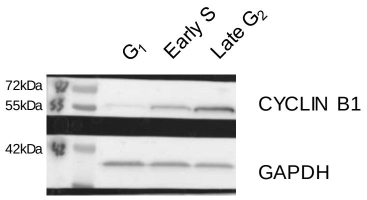

Same samples

Fig. 2A

Exposure time 131,4sec for TRIP12 and SP1:

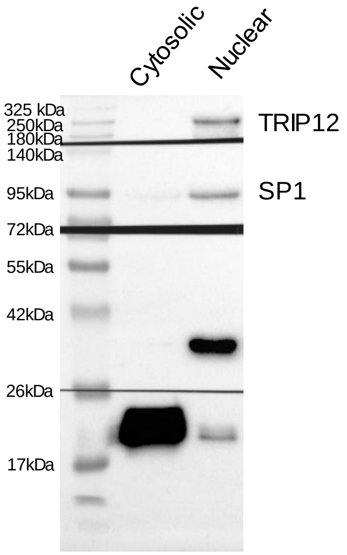

Exposure time 13,1sec for 4EBP1:

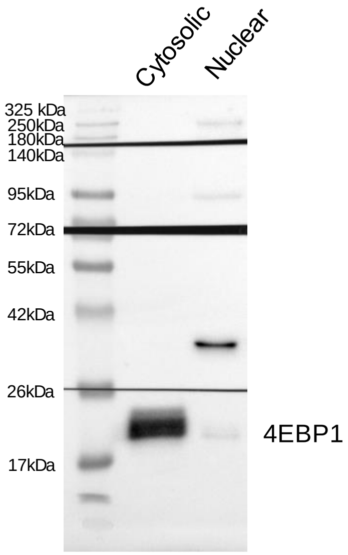

Fig. 2D

Exposure time 19,2sec for TRIP12, SP1 and 4EBP1:

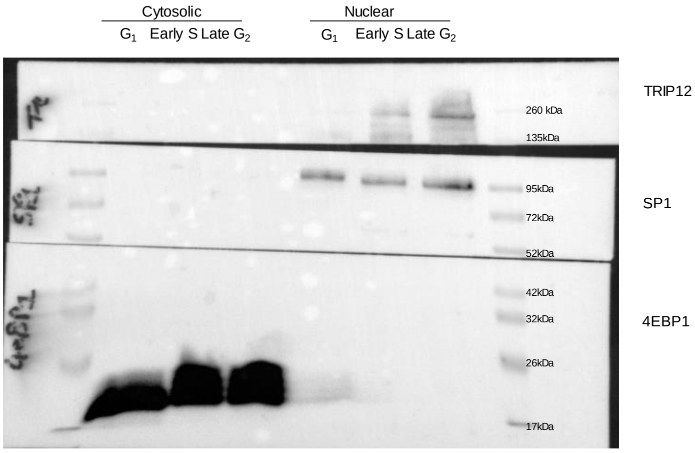

Fig. 2F

Exposure time 10 sec for TRIP12, SP1 and PanH3

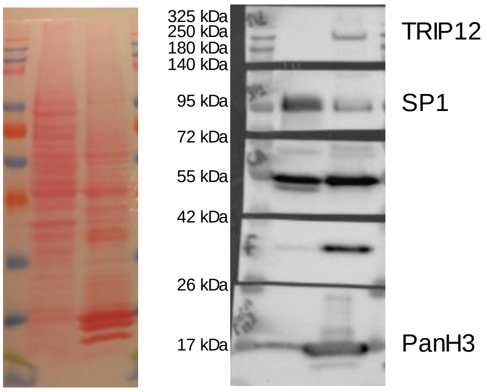

Membrane after
Red Ponceau staining

Exposure time 10 sec for 4EBP1

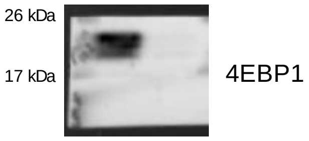

After deblotting the 26-17 kDa strip of the left membrane

Fig. 5A

Exposure time 43,6 sec for TRIP12:

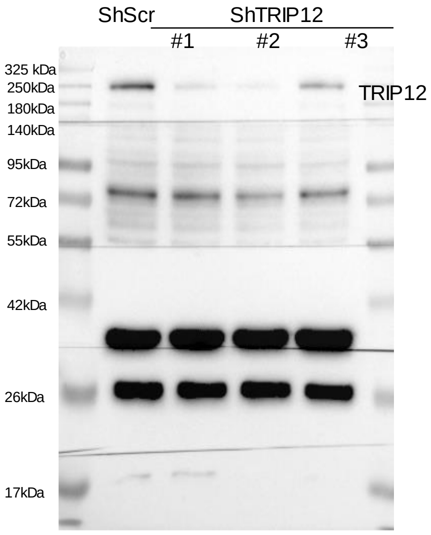

Exposure time 4sec for GAPDH:

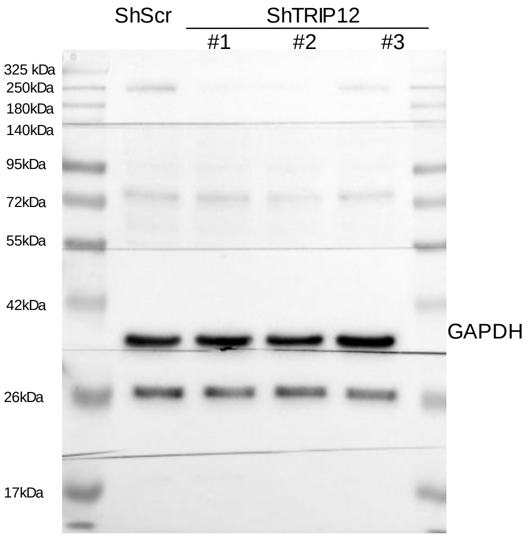

Fig. 6A

Exposure time 1sec for P-Tyr15 CDK1, CDK1, 4EBP1 and panH3 :

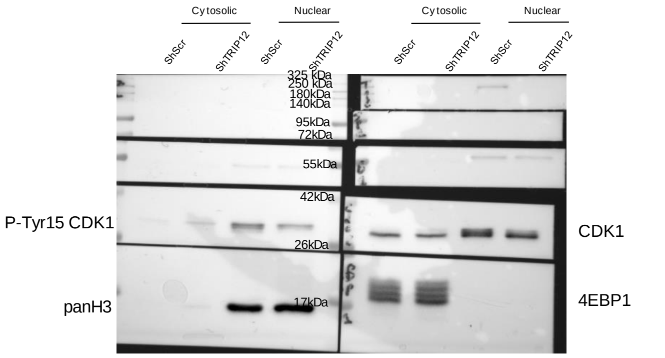

Exposure time 4sec for TRIP12 :

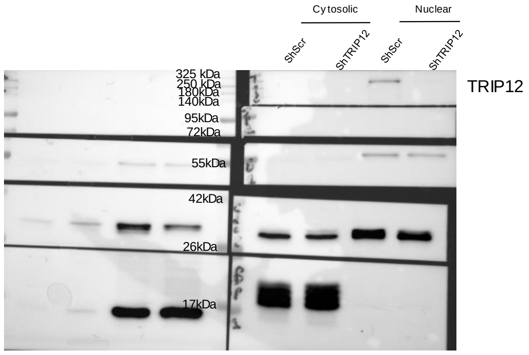

Exposure time 179,9 sec for WEE1 :

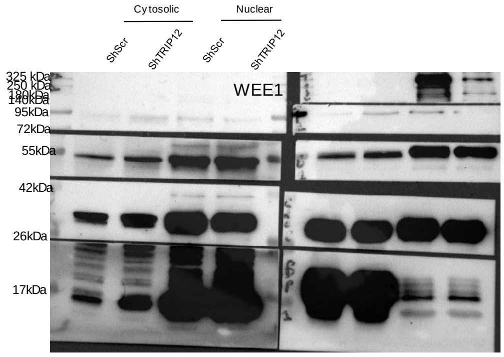

Exposure time 158,7 sec for CDC25C :

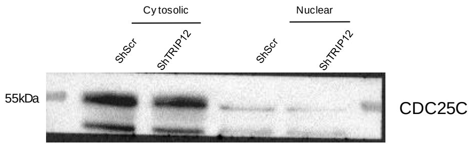

After deblotting the 55kDa strip of the left membrane

Fig.6B

Exposure time 1sec for panH3 and 4EBP1 :

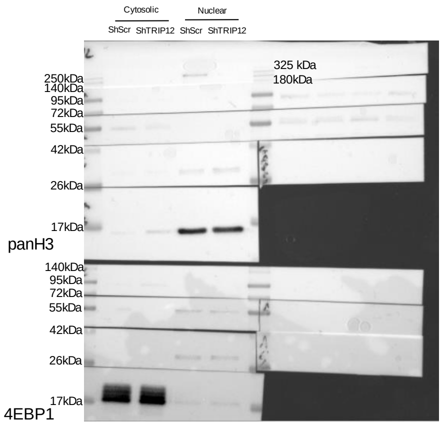

Exposure time 13.1sec for TRIP12 :

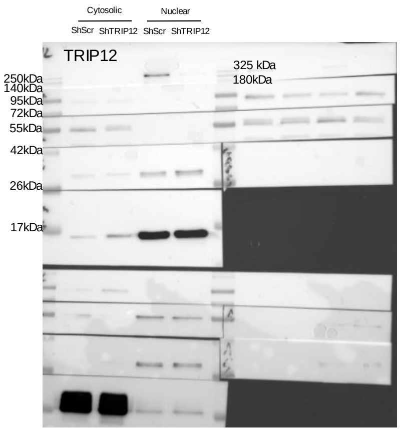

Exposure time 79.9sec for CYCLIN A :

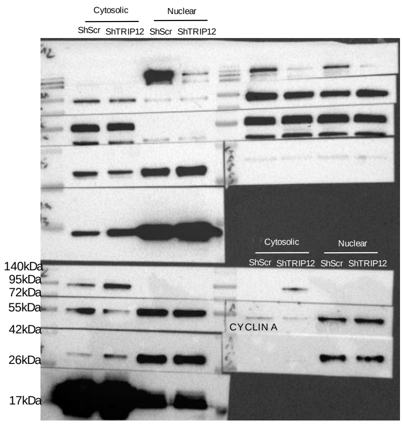

Fig. S6C

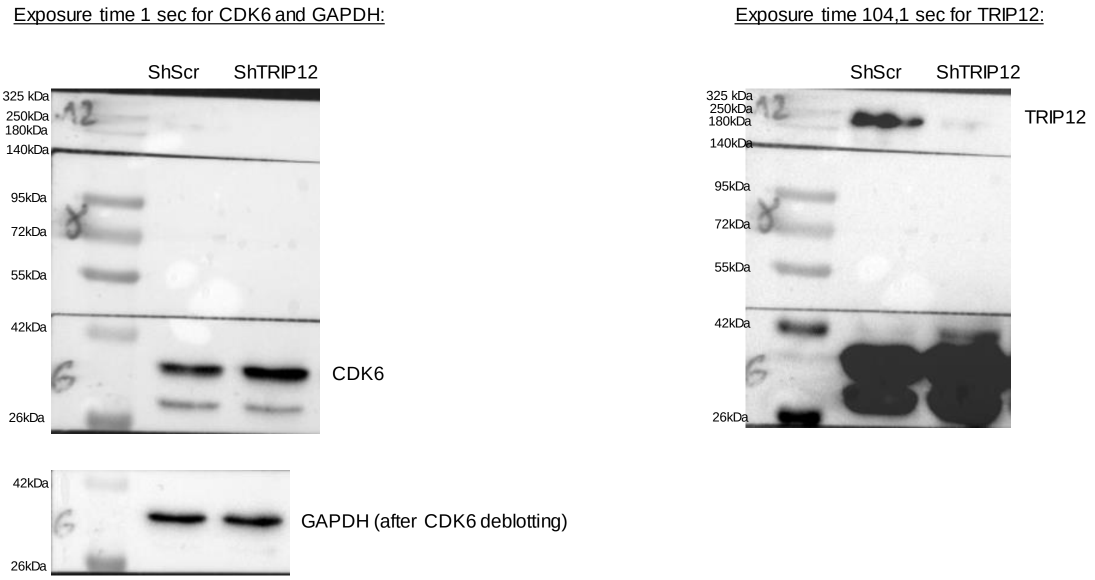
Exposure time 1 sec for CDK6 and GAPDH:
ShScr
ShTRIP12
325 kDa
250kDa
180kDa
140kDa
95kDa
72kDa
55kDa
42kDa
CDK6
26kDa
42kDa
GAPDH (after CDK6 deblotting)
26kDa
Exposure time 104,1 sec for TRIP12:
ShScr
ShTRIP12
325 kDa
250kDa
180kDa
140kDa
TRIP12
95kDa
72kDa
55kDa
42kDa
26kDa
